# Long-term protective immunity induced by an adjuvant-containing live-attenuated AIDS virus

**DOI:** 10.1038/s41541-021-00386-5

**Published:** 2021-10-22

**Authors:** Tomotaka Okamura, Yuya Shimizu, Masamitsu N. Asaka, Tomohiro Kanuma, Yusuke Tsujimura, Takuya Yamamoto, Kazuhiro Matsuo, Yasuhiro Yasutomi

**Affiliations:** 1 Laboratory of Immunoregulation and Vaccine Research, Tsukuba Primate Research Center, National Institutes of Biomedical Innovation, Health and Nutrition, Ibaraki, 305-0843 Japan; 2 Laboratory of Immunosenescence, National Institutes of Biomedical Innovation, Health and Nutrition, Osaka, 567-0085 Japan; 3 Research and Development Department, Japan BCG Laboratory, Tokyo, 204-0022 Japan; 4 Division of Immunoregulation, Department of Molecular and Experimental Medicine, Mie University Graduate School of Medicine, Mie, 514-8507 Japan

**Keywords:** Live attenuated vaccines, HIV infections

## Abstract

The use of an adjuvant in vaccination is thought to be effective for enhancing immune responses to various pathogens. We genetically constructed a live attenuated simian human immunodeficiency virus (SHIV) to express the adjuvant molecule Ag85B (SHIV-Ag85B). SHIV-Ag85B could not be detected 4 weeks after injection in cynomolgus macaques, and strong SHIV-specific T cell responses were induced in these macaques. When the macaques in which SHIV-Ag85B had become undetectable were challenged with pathogenic SHIV89.6P at 37 weeks after SHIV-Ag85B had become undetectable, SHIV89.6P was not detected after the challenge. Eradication of SHIV89.6P was confirmed by adoptive transfer experiments and CD8-depletion studies. The SHIV-Ag85B-inoculated macaques showed enhancement of Gag-specific monofunctional and polyfunctional CD8^+^ T cells in the acute phase of the pathogenic SHIV challenge. The results suggest that SHIV-Ag85B elicited strong sterile immune responses against pathogenic SHIV and that it may lead to the development of a vaccine for AIDS virus infection.

## Introduction

Despite the considerable resources that have been committed to developing an effective human immunodeficiency virus (HIV) vaccine over the past three decades, this objective remains elusive. Although anti-retroviral therapy has led to a dramatic reduction in HIV-related morbidity and mortality, it is not a cure^1^. Recently, there have been two cases in which HIV remission was achieved by cell transplantation^2,3^. The development of an effective vaccine could prevent the spread of HIV infection, but vaccination efforts have been relatively unsuccessful over the past three decades^4,5^. The only successful HIV vaccine to date, evaluated in the RV144 clinical trial, showed an overall efficacy of only 31%^6^.

Various vaccine viruses attenuated by genetic disruption of key regulatory genes including *nef*, *vpx*, *vpr*, and *vif* have been used in previous studies, though the moderately attenuated prototypic vaccine strain SIVmac239∆nef has been used in most studies^7–11^. The live attenuated immunodeficiency viruses *nef*-deleted simian immunodeficiency virus (SIV) and simian human immunodeficiency virus (SHIV) have proven to be highly effective for vaccines in non-human primate models, but they are not sufficiently safe to use as a template for HIV vaccines in humans^12–14^. Several studies have demonstrated that insertion of a cytokine or chemokine gene in live attenuated and genetically defective SIV or SHIV can improve the immunogenicity and enhance the protective capacity of the virus^15–17^ compared to those of safer, less virulent strains.

Antigen 85B (Ag85B) is considered to be an immunogenic protein that can induce a strong T helper type 1 (Th1) immune response in hosts sensitized by Bacillus Calmette–Guérin. Ag85B, which belongs to the Ag85 family, is one of the most dominant proteins secreted from most mycobacterial species. It has been shown to induce substantial Th1 cell proliferation and vigorous Th1 cytokine production in mice^18^. Ag85B has been reported to induce Th1 cells in immunotherapy for atopic dermatitis and allergic asthma^19–21^ and in tuberculosis vaccination^22^. These results make Ag85B an attractive candidate for an immune adjuvant.

Models of SIV infection in non-human primates are important for analysis of acquired immunodeficiency syndrome (AIDS) pathogenesis and determination of the efficacy of an HIV vaccine or therapeutic HIV interventions.　In our previous study, we demonstrated the pathogenesis of SIVmac and that of SHIV89.6P in cynomolgus macaques from Indonesia, Malaysia, and Philippines^23^. A comparison of parameters including plasma viral loads, peripheral CD4^+^ T cell counts, patterns of viral antigen-specific immune responses, and disease outcomes showed that SIV and SHIV were pathogenic in cynomolgus macaques despite the different country origins. Compared to Indian rhesus macaques, cynomolgus macaques from Asian country origins are equally permissive to various strains of SIVmac and SHIV, and they showed longer survival following SIV or SHIV infection, indicating that cynomolgus macaques can provide a model that is close to the course of HIV-1 disease progression in humans.

To study the adjuvant effect of Ag85B in SHIV cynomolgus macaque models, cDNA of *Ag85B* was inserted into the *nef* gene-eliminated site of SHIV, generating SHIV-Ag85B. We then analyzed immune responses in cynomolgus macaques inoculated with SHIV-Ag85B and with parental SHIV-NI and also examined the long-term protective efficacy in those macaques after challenge with pathogenic SHIV89.6P.

## Results

### Construction of SHIV-Ag85B

**Fig. 1:**
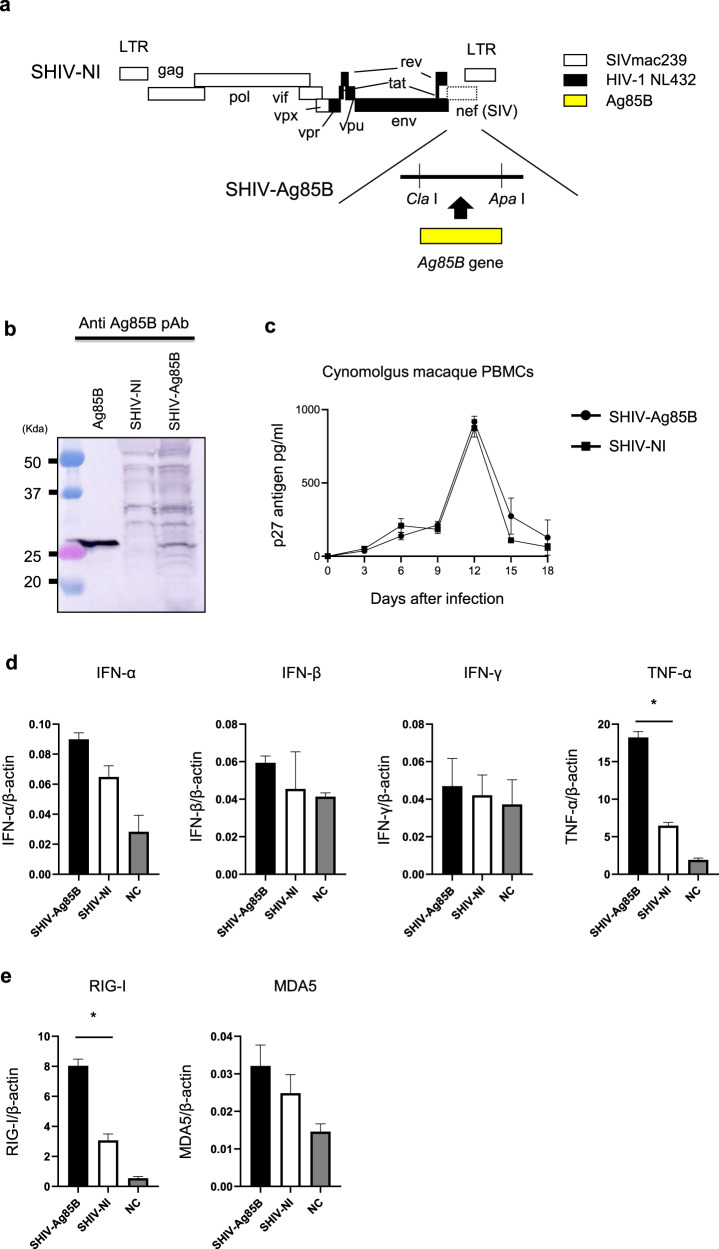
Characterization of SHIV-Ag85B. **a** Genetic structures of SHIV-Ag85B used in this study. SHIV-NI was established by elimination of the *nef* gene of SHIV-MN3rN. SHIV-MN3rN was constructed from HIV-1 NL432 (black region) and SIVmac239 (white region). In SHIV-Ag85B, the *nef* deletion region of SHIV-NI was replaced by the *Ag85B* gene (yellow region). **b** Detection of Ag85B protein by western blotting with an anti-Ag85B polyclonal antibody. **c** Virus replication kinetics of SHIV-Ag85B and SHIV-NI in cynomolgus macaque PBMCs. Representative results of three independent experiments are shown. **d**, **e** Cynomolgus macaque PBMCs were infected with SHIV-Ag85B and SHIV-NI for 48 h, and the increases in mRNA levels of IFN-α, IFN-β, IFN-γ, TNF-α, and RIG-I and MDA5 were determined by real-time PCR. Fold increase of each target gene was normalized to β-actin, and the expression levels are represented as relative values to the control. Control is uninfected cells. Data are averages of triplicate samples from three identical experiments and error bars represent means ± SEM. Statistical analysis were performed using Kruskal–Wallis test. *P < 0.05.

A recombinant SHIV was engineered to express Ag85B in place of *nef* in SHIV-NI (Fig. 1a). Expression of Ag85B was detected by western blot analysis using the cell lysate from a human lymphoid cell line (M8166) infected with SHIV-Ag85B (Fig. 1b). SHIV-ag85B replicated well not only in cynomolgus macaque peripheral blood mononuclear cells (PBMCs) but also in a human lymphoid cell line (CEM×174) (Fig. 1c and supplementary Fig 1a). The replication profile of SHIV-Ag85B in lymphoid cell lines was similar to that of parental SHIV-NI.

### Direct effects of SHIV-Ag85B infection in cells in vitro

We next examined direct effects of SHIV-Ag85B infection such as innate immune responses. Immune responses were assessed at 48 h after infection with SHIV-Ag85B in cynomolgus macaque PBMCs and CEM×174 cells (Fig. 1d, e and supplementary Fig. 1b, c). Expression levels of tumor necrosis factor (TNF)-α after infection with SHIV-Ag85B were significantly higher than those after infection with SHIV-NI. In contrast, mRNA levels of interferon (IFN)-γ and type I IFNs were almost the same after infection with SHIV-Ag85B and after infection with SHIV-NI (Fig. 1d). Double-stranded RNA (dsRNA) is a dominant activator of innate immunity because viral dsRNA is recognized by RIG-I and MDA5^24,25^. The mRNA expression of the intracellular receptor RIG-I was also enhanced by infection with SHIV-Ag85B (Fig. 1e). MDA5 mRNA levels induced by SHIV-Ag85B were similar to those induced by SHIV-NI.

### Viral loads in cynomolgus macaques inoculated with SHIV-Ag85B

**Fig. 2:**
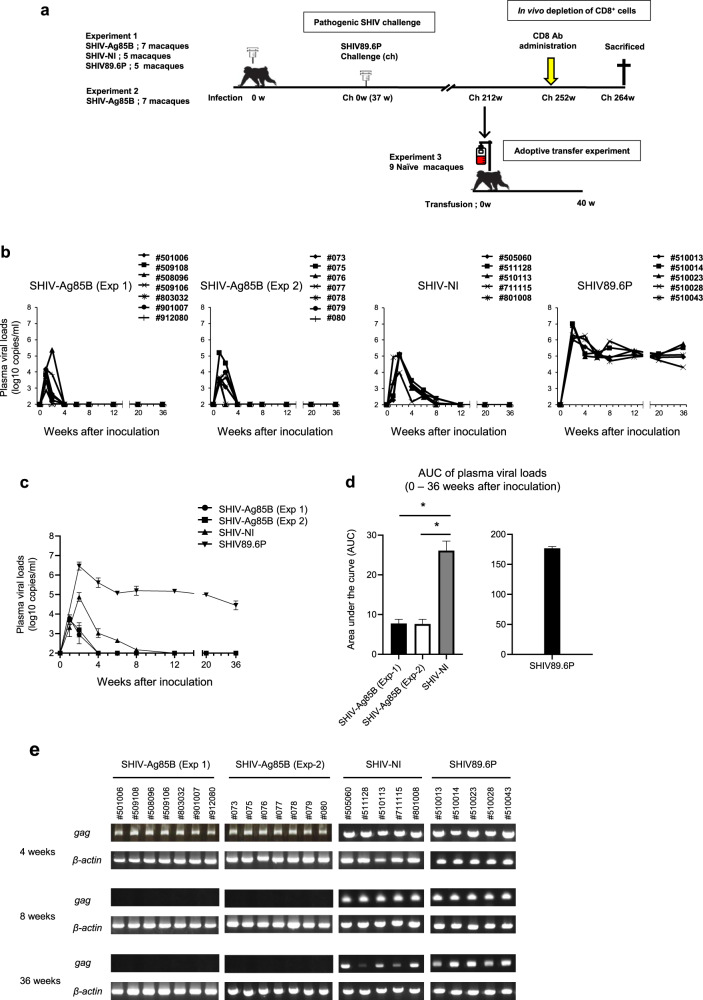
Kinetics of viral loads and detection of *gag* DNA in macaques inoculated with SHIVs. **a** Experimental design, SHIV infection, adoptive transfer experiment, CD8^+^ cell-depletion study, and necropsy time points. **b** Plasma viral loads in macaques inoculated with SHIV-Ag85B (Exp 1), SHIV-Ag85B (Exp 2), SHIV-NI, and SHIV89.6P. Plasma viral loads were measured by quantitative RT-PCR. The detection limit of plasma viral load was 100 copies/ml. **c** Mean plasma viral loads in inoculated macaques. The lines indicate each group means and error bars represent means ± SEM. **d** Area under curve (AUC) of plasma viral loads in inoculated macaques between 0 and 36 weeks post inoculation. Error bars represent means ± SEM. Statistical analysis was performed using Kruskal–Wallis test. *P < 0.05. **e** *Gag* DNA in PBMCs of the macaques after SHIV-Ag85B (Exp 1), SHIV-Ag85B (Exp 2), SHIV-NI, and SHIV89.6P inoculation were detected by nested PCR for the SIV *gag*-specific region. β-Actin was used as a control.

To study the adjuvant effect of Ag85B in SHIV macaque models, two experiments were performed at different times. The design of the macaque study is outlined in Fig. 2a. In the first experiment (Experiment 1 (Exp 1)), all macaques exhibited peak viremia about 2–3 weeks after inoculation (Fig. 2b, c). The plasma viral loads in macaques inoculated with SHIV-Ag85B declined earlier than those in macaques inoculated with SHIV-NI. The plasma viral load in macaques inoculated with SHIV-Ag85B fell below the measurable limit at 2–4 weeks after inoculation, whereas that in macaques inoculated with SHIV-NI reached an undetectable level at about 8–12 weeks after inoculation (Fig. 2b, c). In contrast, the plasma viral loads in macaques inoculated with SHIV89.6P were maintained at high levels after the acute phase of inoculation (Fig. 2b, c). The area under the curve (AUC) of viral loads in SHIV-Ag85B-inculated macaques was significantly smaller than that in SHIV-NI- or SHIV89.6P-inoculated macaques (Fig. 2d). Due to limited viral replication, *gag* products in SHIV-Ag85B-inoculated macaques were detected up to 4 weeks after inoculation and became undetectable in SHIV-Ag85B-inoculated macaques at 8 weeks after inoculation (Fig. 2e). The stability of the inserted *Ag85B* gene in SHIV-Ag85B was analyzed by PCR. The full length of the inserted *Ag85B* gene in PBMCs from macaques inoculated with SHIV-Ag85B was detected 2 weeks after inoculation (Supplementary Fig. 2). SHIV-NI- and SHIV89.6P-inoculated macaques showed *gag* products in PBMCs examined at all stages of infection (Fig. 2e). After the inoculation, peripheral blood CD4^+^ T cells remained within the normal range of levels in both SHIV-Ag85B- and SHIV-NI-inoculated macaques, and these macaques remained healthy clinically (Supplementary Fig. 3). In contrast, SHIV89.6P-inoculated macaques showed very low CD4^+^ T cell counts (<150 cells/μl) during the observation period (Supplementary Fig. 3). In the second experiment (Experiment 2 (Exp 2)), all of the macaques inoculated with SHIV-Ag85B showed viremia within 2 weeks after inoculation (Fig. 2b, c). In these macaques, plasma viral RNAs and proviral DNAs were reduced to almost undetectable levels within 4–8 weeks after inoculation and the numbers of peripheral CD4^+^ T cells were maintained at normal levels (Fig. 2b–e and Supplementary Fig. 3). SHIV-Ag85B infection was confirmed in all experimental animals for at least up to 4 weeks after inoculation; however, SHIV-Ag85B might have been eradicated from macaques at 8 weeks after inoculation.

### SHIV-Ag85B-induced antigen-specific CD8^+^ immune response

**Fig. 3:**
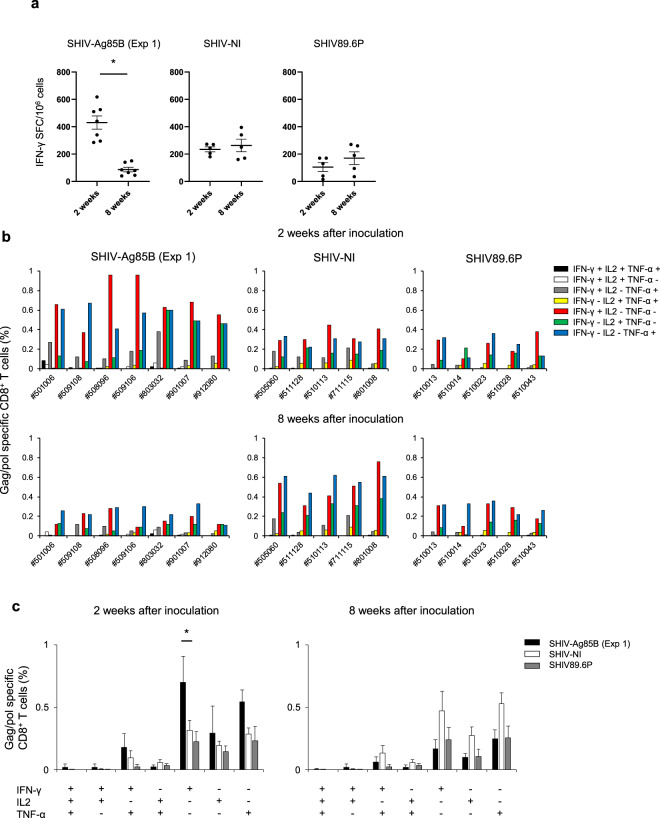
SHIV antigen-specific T cell responses in macaques inoculated with SHIVs. **a** Numbers of Gag/pol-specific IFN-γ-producing cells in macaques inoculated with SHIV-Ag85B (Exp 1), SHIV-NI, and SHIV89.6P were determined by ELISPOT assays. PBMCs obtained at 2 and 8 weeks after SHIV inoculation were co-cultured for 6 h with autologous B-LCL cells that had been infected with a recombinant vaccinia virus expressing SIV Gag/pol. Antigen-specific IFN-γ ELISPOT results are depicted as spots per 10^6^ PBMCs. Each symbol shows one animal, and error bars represent means ± SEM. Statistical analysis was performed using Wilcoxon signed-rank test. *P < 0.05. **b** Percentages of Gag/pol-specific CD8^+^ T cells producing IFN-γ, TNF-α, and IL-2 in macaques inoculated with SHIV-Ag85B (Exp 1), SHIV-NI, and SHIV89.6P.　The cytokine profile in cells was determined by flow cytometry by gating for lymphocytes and CD8^+^ T cells. PBMCs obtained at 2 and 8 weeks after SHIV inoculation were co-cultured for 6 h with autologous B-LCL cells that had been infected with a recombinant vaccinia virus expressing SIV Gag/pol. **c** Mean percentages of Gag/pol-specific induction of single or multiple cytokines in macaques inoculated with SHIV-Ag85B (Exp 1), SHIV-NI, and SHIV89.6P. Error bars represent means ± SEM. Statistically significant differences between SHIV-Ag85B (Exp 1) versus SHIV-NI were determined by using Student’s *t* test. *P < 0.05.

SHIV antigen (Gag/pol)-specific T cell responses were measured by an IFN-γ ELISPOT assay of PBMCs. The IFN-γ ELISPOT responses to Gag/pol in SHIV-Ag85B-inoculated macaques at 2 weeks after inoculation were stronger than those in other groups of animals, and the responses were decreased at 8 weeks after inoculation (Fig. 3a). We also analyzed Ag85B-specific IFN-γ ELISPOT responses of PBMCs from SHIV-Ag85B-inoculated macaques at 2 weeks after inoculation. These macaques showed weak immune responses to Ag85B in ELISPOT analysis (Supplementary Fig. 4). IFN-γ ELISPOT responses to Gag/pol were detected in SHIV-NI-inoculated macaques at 2 weeks and were slightly increased at 8 weeks after inoculation. The macaques inoculated with SHIV89.6P showed weak immune responses at all time points in ELISPOT analysis (Fig. 3a). To determine whether polyfunctional Gag/pol-specific CD8^+^ T cell immune responses were induced in SHIV-Ag85B-inoculated macaques, we analyzed intracellular cytokine staining (ICS) for IFN-γ, TNF-α, and interleukin-2 (IL-2) by flow cytometry. SHIV-Ag85B-inoculated macaques showed preferential accumulation of Gag/pol-specific IFN-γ or TNF-α-single-positive and IFN-γ and TNF-α-double-positive CD8^+^ T cells in PBMCs at 2 weeks after inoculation, and these responses were decreased at 8 weeks after inoculation (Fig. 3b, c). SHIV-Ag85B also elicited antigen-specific CD4^+^ T cells producing a single cytokine (Supplementary Fig. 5). In the SHIV-NI-inoculated macaques, the percentages of antigen-specific CD8^+^ T cells and CD4^+^ T cells were lower than those in SHIV-Ag85B-inoculated macaques at 2 weeks after inoculation; however, the percentages of those cells in SHIV-NI-inoculated macaques were higher than those in SHIV-Ag85B-inoculated macaques at 8 weeks after inoculation. In the SHIV89.6P-inoculated macaques, the percentages of those cells were lower at all time points (Fig. 3b, c and Supplementary Fig. 5).

SHIV Env-specific antibody titers in plasma of SHIV-Ag85B, SHIV-NI, and SHIV89.6P-inoculated macaques were examined by an enzyme-linked immunosorbent assay (ELISA). All of the macaques showed antibody responses against HIV Env at 4–8 weeks after inoculation (Supplementary Fig. 6). In the SHIV-NI-inoculated macaques, anti-HIV Env antibody titers were higher than those in SHIV-Ag85B- or SHIV-89.6P-inoculated macaques during the observation period (Supplementary Fig. 6).

### Challenge with a pathogenic SHIV89.6P

**Fig. 4:**
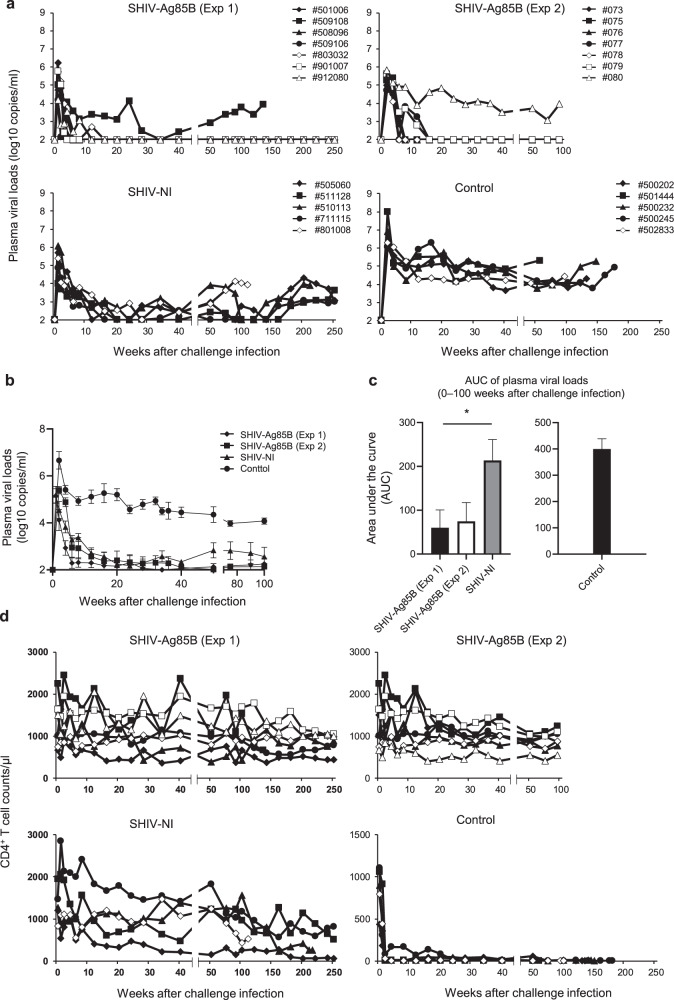
Kinetics of viral loads, CD4^+^ T cells, and proviral DNA in macaques inoculated with SHIV-Ag85B or SHIV-NI after pathogenic SHIV89.6P challenge. **a** Plasma viral loads in macaques inoculated with SHIV-Ag85B (Exp 1), SHIV-Ag85B (Exp 2), and SHIV-NI after SHIV89.6P challenge. As controls, 5 naive macaques were challenged intravenously with SHIV89.6P. Plasma viral loads were measured by quantitative RT-PCR. The detection limit of plasma viral load was 100 copies/ml. **b** Mean plasma viral loads in each group after SHIV challenge. The lines indicate each group means and error bars represent means ± SEM. **c** Area under curve (AUC) of plasma viral loads in each group between 0 and 100 weeks post challenge infection. Error bars represent means ± SEM. Statistical analysis was performed using Kruskal–Wallis test. *P < 0.05. **d** Count of CD4^+^ T cells was analyzed by flow cytometry and whole blood cell counts. Whole blood was stained by CD3, CD4, and CD8 Abs, and CD4^+^ T cell counts were determined by flow cytometric analysis.

To know the effect of pathogenic virus infection in SHIV-Ag85B- or SHIV-NI-inoculated macaques, all of the macaques were challenged intravenously with 10^5^ tissue culture 50% infectious dose (TCID_50_) of pathogenic SHIV89.6P at 37 weeks after inoculation. The peak plasma viral loads in SHIV-Ag85B-inoculated macaques were similar to those in SHIV-NI-inoculated macaques (Fig. 4a, b). The plasma viral loads in six of the seven SHIV-Ag85B-inoculated macaques declined to almost below the detection level from 6 to 16 weeks after the challenge; however, viral RNAs remained negative for >250 weeks (in the chronic phase) in six macaques. In the SHIV-Ag85B-inoculated macaques, AUC values of viral loads from 0 to 100 weeks after the challenge were smaller than those in SHIV-NI-inoculated macaques (Fig. 4c). Moreover, CD4^+^ T cells in SHIV-Ag85B-inoculated macaques were maintained at the normal level (Fig. 4d). In addition, six of the seven SHIV-Ag85B-inoculated macaques did not show progression to obvious AIDS-like disease. In the second experiment, six of the seven SHIV-Ag85B-inoculated macaques did not show any detectable plasma viral load from 6 to 20 weeks after the challenge (Fig. 4a, b). In the chronic phase, viral RNAs remained negative for >20 weeks in six macaques. The AUC values of viral loads after the challenge were not significantly different for SHIV-Ag85B (Exp 1) and SHIV-Ag85B (Exp 2) (Fig. 4c). The number of CD4^+^ T cells was maintained at the normal level in all of these macaques (Fig. 4d). In contrast, the plasma viral loads of two SHIV-Ag85B-inoculated macaques (#509108 and #080) were similar to those of SHIV-NI-inoculated macaques. CD4^+^ T cells in one macaque (#509108) gradually decreased during the observation period. That macaque (#509108) showed AIDS symptoms at 989 days after the challenge. In the SHIV-NI-inoculated macaques, the set-point plasma viral loads were <10^4^ copies/ml after the acute phase (Fig. 4a, b). CD4^+^ T cells in these macaques gradually decreased during the observation period (Fig. 4d). Thereafter, two of the SHIV-NI-inoculated macaques (#510113 and #801008) showed AIDS symptoms at 718 and 1450 days after the challenge, respectively. In the control macaques, viremia was maintained at high levels during the observation period (Fig. 4a, b). These macaques showed very low CD4^+^ T cell counts (<100 cells/μl) during the observation period (Fig. 4d) and showed AIDS symptoms at 402–1225 days after the challenge.

### Quantification of proviral DNA after pathogenic SHIV89.6P challenge

**Fig. 5:**
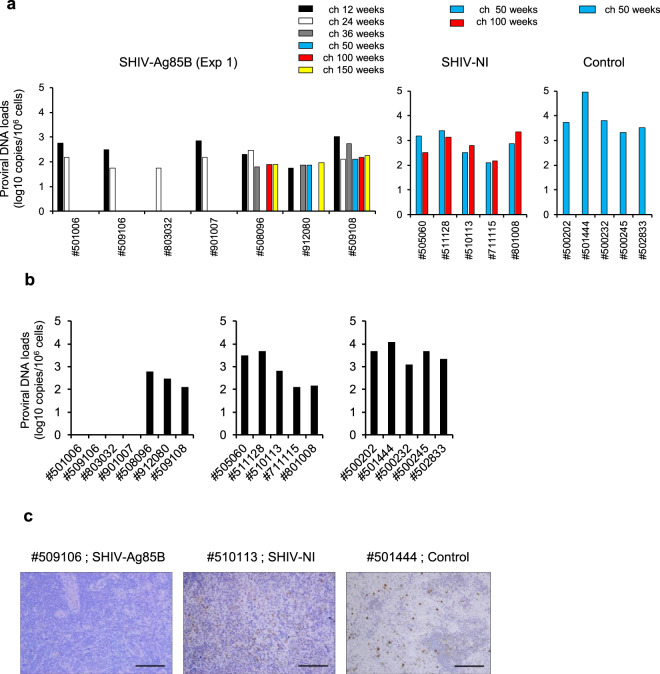
Kinetics of proviral DNA in macaques inoculated with SHIV-Ag85B or SHIV-NI after pathogenic SHIV89.6P challenge. **a** Proviral DNA loads in PBMCs of SHIV-Ag85B-inoculated macaques (Exp 1) or SHIV-NI-inoculated macaques after SHIV89.6P challenge as measured by ultrasensitive digital PCR. The detection limit of proviral DNA load was 5 copies per 10^6^ cells. **b** Proviral DNA loads in lymphoid tissues of SHIV-Ag85B-inoculated macaques (Exp 1) or SHIV-NI-inoculated macaques at 34 weeks after the challenge as measured by ultrasensitive digital PCR. **c** Immunohistochemical detection of SHIV Nef antigen in inguinal lymph nodes at 34 weeks after the challenge. Brown staining indicates SHIV Nef-positive cells.　Magnification ×200. Black scale bars, 100 μm.

To better understand the viral status in SHIV-Ag85B-inoculated macaques (#501006, #509106, #803032, #901007, #508096, #912080, and #509108), proviral DNAs in PBMCs and lymphoid tissues after the SHIV89.6P challenge were measured by ultrasensitive digital PCR. All SHIV-Ag85B-inoculated macaques showed proviral DNA loads in PBMCs at 12 or 24 weeks after the challenge (Fig. 5a). Strikingly, in a period of 36 weeks after the challenge, four of the SHIV-Ag85B-inoculated macaques (#501006, #509106, #803032 and #901007) did not exhibit any proviral DNA of SHIV in PBMCs, and proviral DNA in PBMCs remained negative for >50 weeks in four macaques (Fig. 5a). Moreover, proviral DNA was not detected in lymphoid tissues of those macaques (Fig. 5b). Also, SIV Nef-positive cells were not detected by immunohistochemistry in lymphoid tissues from four macaques (Fig. 5c). Two SHIV-Ag85B-inoculated macaques (#508096 and #912080) without a plasma viral load showed proviral DNAs in PBMCs and lymphoid tissues during the observation period (Fig. 5a, b). Control macaques and the SHIV-NI-inoculated macaques showed high levels of proviral DNAs in PBMCs and lymphoid tissues after the SHIV-89.6P challenge (Fig. 5a, b).

### Protective antigen-specific immune responses after pathogenic SHIV89.6P challenge

Four of the seven SHIV-Ag85B-inoculated macaques (#501006, #509106, #803032, and #901007) showed no increase in overall plasma viral RNA and proviral DNA, and small amounts of proviral DNA were detected in two macaques (#508096 and #912080) in the SHIV-Ag85B-inoculated macaques. One SHIV-Ag85B-inoculated macaque (#509108) showed a low level of plasma viral RNA that was similar to the levels in five SHIV-NI-inoculated macaques (#505060, #511128, #510113, #711115, and #801008). Those 6 macaques (1 SHIV-Ag85B-inoculated macaque and 5 SHIV-NI-inoculated macaques) showed low levels of plasma viral RNA compared to the levels in control macaques, and 5 SHIV89.6P-injected naive macaques (#500202, #501444, #500232, #500245, and #502833) showed stable viremia above 10^4^–10^7^ copies/ml of viral RNA (Figs. 4a and 5a).

**Fig. 6:**
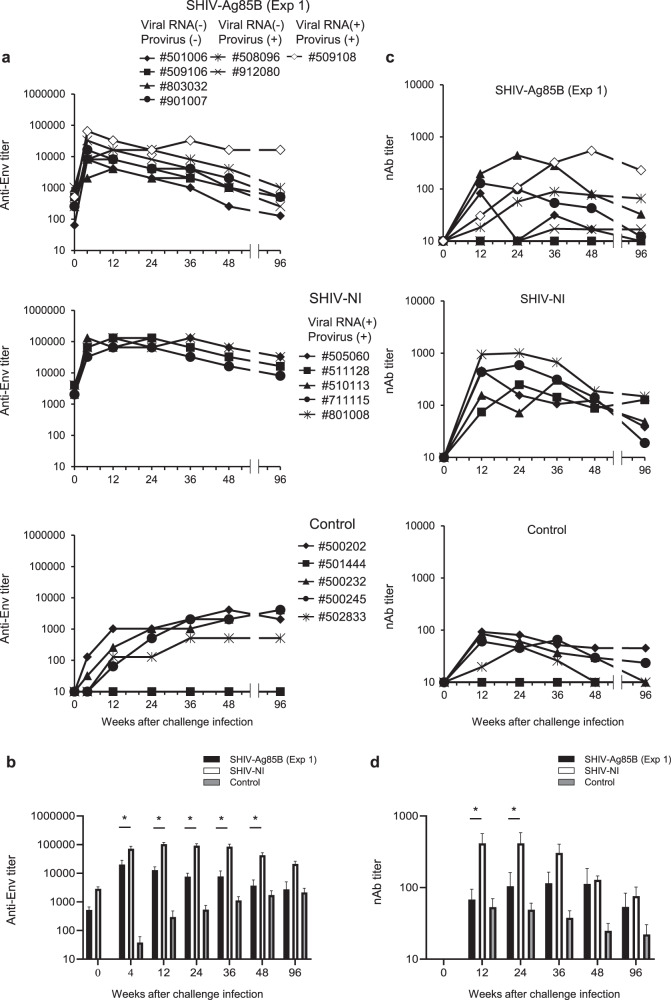
SHIV antigen-specific antibody responses after pathogenic SHIV89.6P challenge. **a** Env-specific antibody immune responses after SHIV89.6P challenge. Kinetics of antibody titers in SHIV-Ag85B (Exp 1)-inoculated macaques, SHIV-NI-inoculated macaques, and control macaques after the SHIV89.6P challenge were measured by ELISA at each time point. **b** Means of antigen-specific antibody titers in SHIV-Ag85B (Exp 1)-inoculated macaques, SHIV-NI-inoculated macaques, and control macaques after SHIV89.6P challenge. Error bars represent means ± SEM. Statistical analysis was performed using two-way analysis of variance with Tukey’s test. *P < 0.05. **c** Neutralizing antibodies (nAb) against SHIV89.6P in SHIV-Ag85B (Exp 1)-inoculated macaques, SHIV-NI-inoculated macaques and control macaques after the challenge. Serially diluted plasma from SHIV-Ag85B (Exp 1)-inoculated macaques, SHIV-NI-inoculated macaques, and control macaques was used　to determine　the　lowest　reciprocal dilution　that　results　in　50%　reduction　of　SHIV89.6P infectivity in TZM-bl assays. **d** Means of neutralizing antibodies titers in SHIV-Ag85B (Exp 1)-inoculated macaques, SHIV-NI-inoculated macaques, and control macaques after SHIV89.6P challenge. Error bars represent means ± SEM. Statistical analysis was performed using two-way analysis of variance with Tukey’s test. *P < 0.05.

After the pathogenic SHIV89.6P challenge, we measured the HIV Env-specific antibody levels in plasma of SHIV-Ag85B-inoculated macaques, SHIV-NI-inoculated macaques, and control macaques. Antibody titers in SHIV-Ag85B- and SHIV-NI-inoculated macaques rapidly increased and reached peak levels within 4 weeks after the challenge (Fig. 6a, b). In the six SHIV-Ag85B-inoculated macaques, anti-SHIV antibody titers then gradually decreased during the observation period. In contrast, one SHIV-Ag85B-inoculated macaque (#509108) and five SHIV-NI-inoculated macaques maintained strong antibody responses (Fig. 6a, b). Neutralizing antibody titers in plasma directed against SHIV89.6P were monitored longitudinally (Fig. 6c, d). On the day of the pathogenic SHIV89.9 challenge, we did not detect neutralizing antibodies against SHIV-89.6P in any of the macaques. Six of the seven SHIV-Ag85B-inoculated macaques developed low levels of neutralizing antibody titers (Fig. 6c, d). Only one macaque (#509108) developed increasing neutralization responses against the challenge virus. In SHIV-NI-inoculated macaques, neutralizing antibodies against SHIV89.6P were detected at 12 weeks after the challenge, and then most of the macaques maintained the neutralizing antibody responses. On the other hand, control macaques developed low levels of neutralizing antibody titers (Fig. 6c, d).

**Fig. 7:**
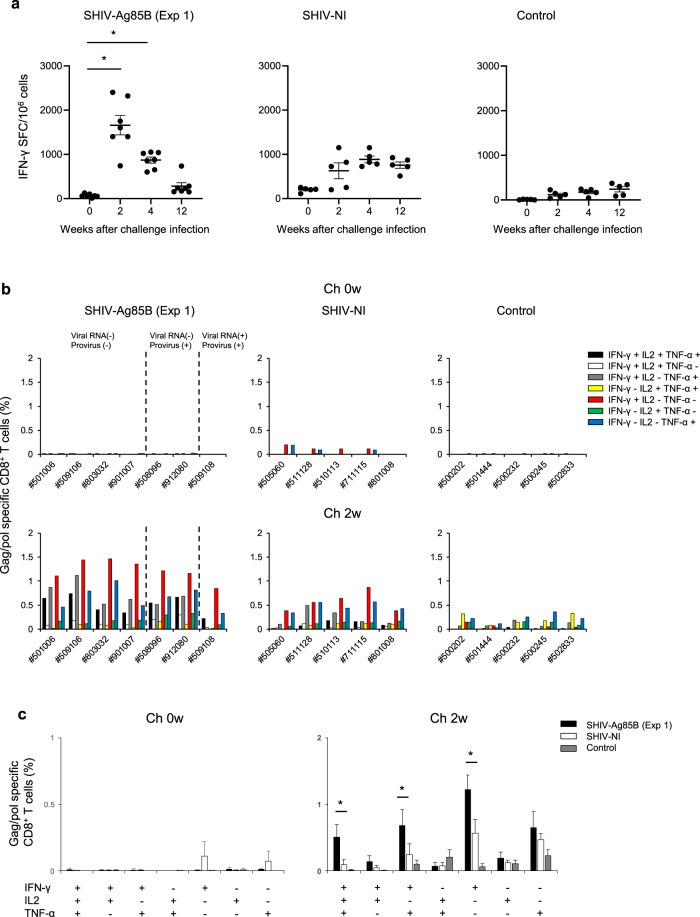
Protective antigen-specific T cell responses after pathogenic SHIV89.6P challenge. **a** Kinetics of Gag/pol-specific IFN-γ-producing cells in SHIV-Ag85B (Exp 1)-inoculated macaques, SHIV-NI-inoculated macaques, and control macaques after SHIV89.6P challenge was determined by ELISPOT assays. PBMCs obtained at 0, 2, 4, and 12 weeks after the challenge were co-cultured for 9 h with autologous B-LCL cells that had been infected with a recombinant vaccinia virus expressing SIV Gag/pol. Antigen-specific IFN-γ ELISPOT results are depicted as spots per 10^6^ PBMCs. Each symbol shows one animal, and error bars represent means ± SEM. Statistical analysis was performed using Wilcoxon signed-rank test. *P < 0.05. **b** Percentages of Gag/pol-specific CD8^+^ T cells producing IFN-γ, TNF-α, and IL-2 in SHIV-Ag85B (Exp 1)-inoculated macaques, SHIV-NI-inoculated macaques, and control macaques after SHIV89.6P challenge. The cytokine profile in cells was determined by flow cytometry by gating for lymphocytes and CD8^+^ T cells. PBMCs obtained at 0 and 2 weeks after the challenge were co-cultured for 6 h with autologous B-LCL cells that had been infected with a recombinant vaccinia virus expressing SIV Gag/pol. **c** Mean percentages of Gag/pol-specific induction of single or multiple cytokines in SHIV-Ag85B (Exp 1)-inoculated macaques, SHIV-NI-inoculated macaques, and control macaques after SHIV89.6P challenge. Error bars represent means ± SEM. Statistically significant differences between SHIV-Ag85B (Exp 1) and SHIV-NI were determined by using Student’s *t* test. *P < 0.05.

We next analyzed SHIV antigen-specific production of IFN-γ by ELISPOT assays in these experimental macaques after the challenge. In six SHIV-Ag85B-inoculated macaques, ELISPOT responses were increased dramatically at 2 weeks after the challenge (Fig. 7a). The responses were reduced in most of those macaques at 12 weeks after the challenge. In only one of the SHIV-Ag85B-inoculated macaques, the IFN-γ ELISPOT response was moderately increased at 2 weeks after the challenge and was maintained at 12 weeks after the challenge. The response was similar to ELISPOT responses of SHIV-NI. The control macaques showed weak IFN-γ ELISPOT responses at all time points (Fig. 7a). The polyfunctionality of Gag/pol-specific T cells was also examined by flow cytometry. On the day of the challenge, we did not detect polyfunctional Gag/pol-specific CD4^+^ and CD8^+^ T cells in any of the macaques (Fig. 7b and Supplementary Fig. 7). In six SHIV-Ag85B-inoculated macaques, there was preferential accumulation of Gag/pol-specific, triple-positive (IFN-γ, TNF-α and IL-2), and IFN-γ or TNF-α-single-positive CD8^+^ T cells at 2 weeks after the challenge (Fig. 7b, c). In addition, the percentages of these polyfunctional Gag/pol-specific CD8^+^ T cells in SHIV-Ag85B-inoculated macaques were significantly higher than those in SHIV-NI-inoculated macaques (Fig. 7b, c). We found polyfunctional Gag/pol-specific CD4^+^ T cells in six SHIV-Ag85B-inoculated macaques, but the percentages were similar to those in SHIV-NI-inoculated macaques at 2 weeks after the challenge (Supplementary Fig. 7). We also analyzed the memory phenotype of SHIV antigen-specific CD8^+^ T cells in those three groups. The percentages of Gag/pol-specific IFN-γ-producing CD8^+^ T cells in six SHIV-Ag85B-inoculated macaques were higher than those in SHIV-NI-inoculated macaques and control macaques (Supplementary Fig. 8). In those macaques, Gag/pol-specific responses were a mixed effector (EM) and central memory (CM) phenotype for CD8^+^ T cells (Supplementary Fig. 8).

### Inverse correlation between viral load and SHIV antigen-specific T cell responses in SHIV-Ag85B-inoculated macaques

**Fig. 8:**
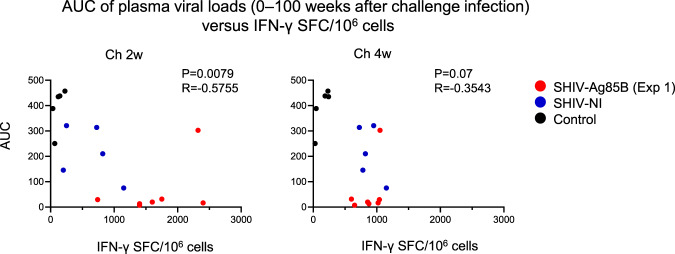
Inverse correlation analyses between protective T cell responses and AUC of plasma viral loads. Analysis of correlation between Gag/pol-specific IFN-γ-producing cells at 2 or 4 weeks post challenge infection and AUC of plasma viral loads. AUC was inversely correlated with Gag/pol-specific IFN-γ-producing cells at 2 weeks post challenge infection (*p* = 0.0079, *r* = −0.5755). No significant correlation was observed between AUC and Gag/pol-specific IFN-γ-producing cells at 4 weeks post challenge infection. Statistical analyses were performed using Spearman correlation.

We assessed whether strong Gag/pol-specific T cell responses were associated with viral loads in SHIV-Ag85B-inoculated macaques after the challenge (Fig. 8). The Gag/pol-specific IFN-γ-producing cells at 2 weeks after challenge infection was inversely correlated with AUC of plasma viral loads. An inverse correlation, however, was not found for Gag/pol-specific IFN-γ-producing cells at 4 weeks after challenge infection (Fig. 8). These results suggest that rapidly induced SHIV antigen-specific T cell responses resulted in a reduction of plasma viral loads in SHIV-Ag85B-inoculated macaques after the challenge.

### Eradication of pathogenic SHIV89.6P in SHIV-Ag85B-inoculated macaques

**Fig. 9:**
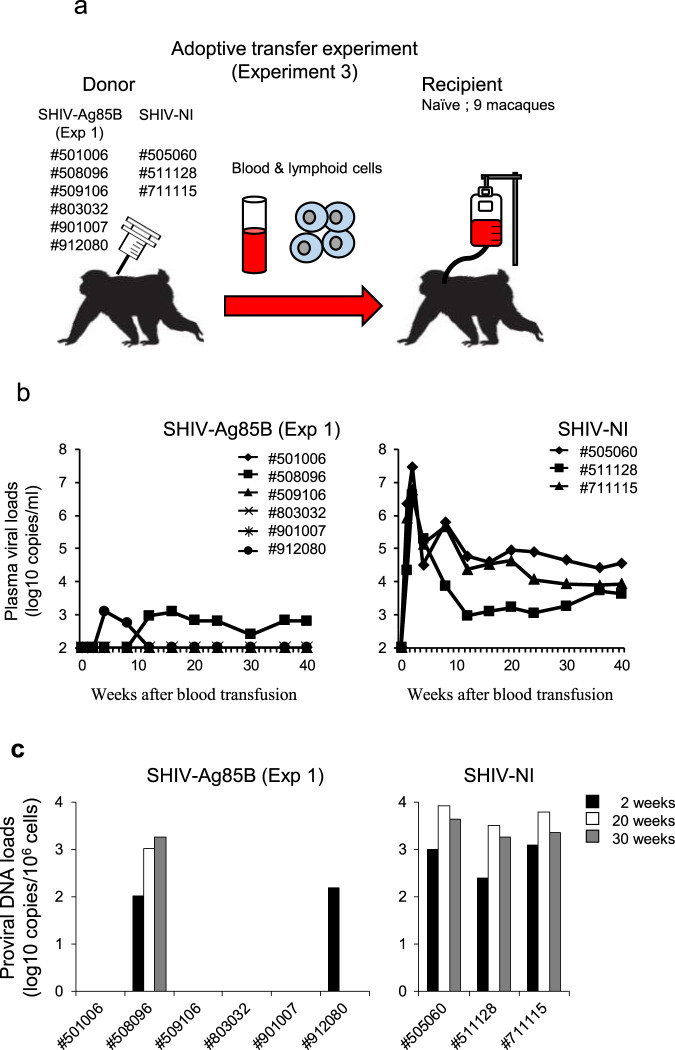
Kinetics of viral loads and proviral DNA after adoptive transfer. **a** Schematic of the adoptive transfer experiment (Experiment 3). **b** Plasma viral loads following adoptive transfer are shown. Plasma viral loads were measured by quantitative RT-PCR. The detection limit of plasma viral load was 100 copies/ml. **c** Proviral DNA loads in PBMCs following adoptive transfer are shown. Proviral DNA loads were measured by ultrasensitive digital PCR. The detection limit of proviral DNA load was 5 copies per 10^6^ cells.

To examine the eradication of virus in the animals, we performed adoptive transfer experiments (Experiment 3) and infused peripheral blood and lymph node mononuclear cells by the intravenous route from six SHIV-Ag85B-inoculated macaques (#501006, #508096, #509106, #803032, #901007, and #912080) and three SHIV-NI-inoculated macaques (#505060, #511128 and #711115) into naive cynomolgus macaques (Fig. 9a). Adoptive transfer of cells from all SHIV-NI-inoculated macaques at 212 weeks after the challenge readily transferred typical SHIV infection to naive macaques. In addition, adoptive transfer of cells from two SHIV-Ag85B-inoculated macaques (#508096 and #912080) at 212 weeks after the challenge readily transferred infection to naive macaques and the macaques showed plasma viral RNA and proviral DNA, indicating the presence of replicating virus in those macaques (Fig. 9b, c). In contrast, adoptive transfer of cells from four SHIV-Ag85B-inoculated macaques (#501006, #509106, #803032, and #901007) at 212 weeks after the challenge did not transfer SHIV infection to naive macaques (Fig. 9b, c), indicating that cells containing pathogenic virus were not present in these cell preparations from SHIV-Ag85B- and SHIV-89.6P-inoculated macaques.

**Fig. 10:**
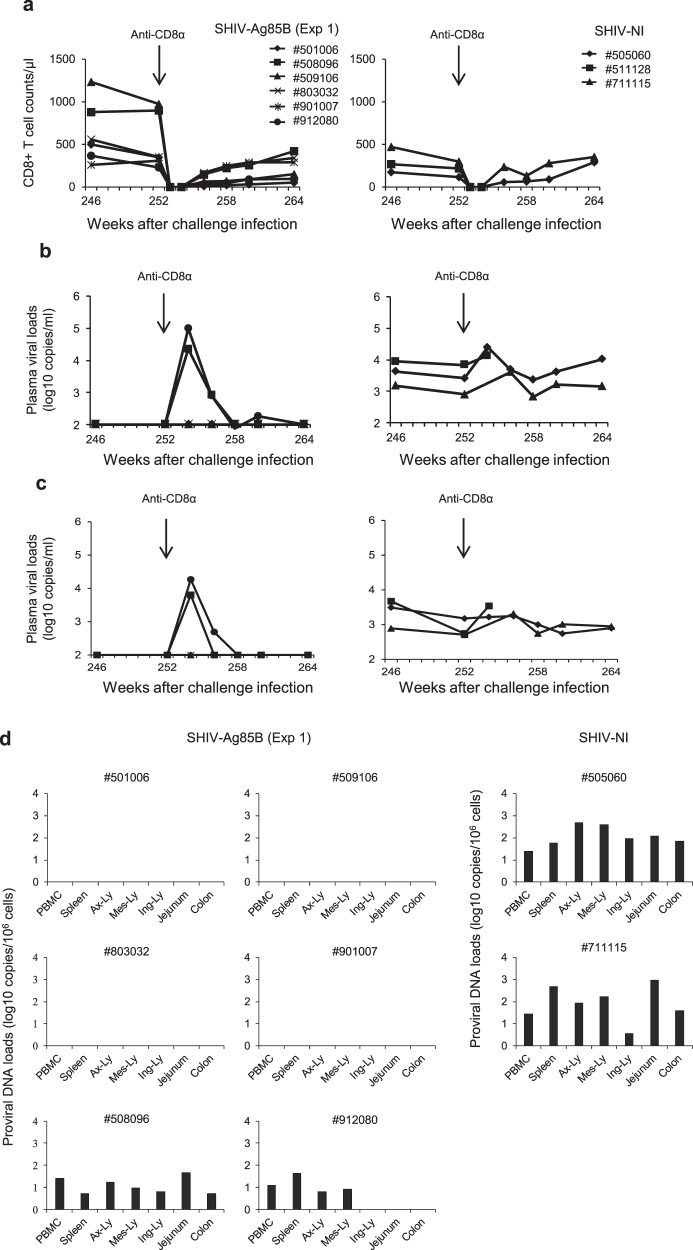
Virology analysis in SHIV-Ag85B-mediated protection following CD8^+^ cell depletion. **a** Changes in peripheral CD8^+^ T cell counts after anti-CD8 antibody administration. SHIV-Ag85B (Exp 1)-inoculated macaques and SHIV-NI-inoculated macaques were administered an anti-CD8^+^ antibody at a dose of 15 mg per kg body weight at 252 weeks after SHIV89.6P challenge. **b** Kinetics of plasma viral RNA loads after administration of the anti-CD8 antibody. Plasma viral loads were measured by quantitative RT-PCR. The detection limit of plasma viral load was 100 copies/ml. **c** Kinetics of SHIV89.6P-specific plasma viral RNA loads after administration of the anti-CD8 antibody. Plasma viral loads were measured by quantitative RT-PCR. The detection limit of plasma viral load was 1000 copies/ml. **d** At necropsy, SHIV proviral DNA loads in PBMCs, the spleen, axillary lymph node (Ax-Ly), mesenteric lymph node (Mes-Ly), inguinal lymph node (Ing-Ly), jejunum, and colon of SHIV-Ag85B (Exp 1)-inoculated macaques and SHIV-NI-inoculated macaques were measured by ultrasensitive digital PCR. The detection limit of proviral DNA load was 5 copies per 10^6^ cells.

We next depleted CD8^+^ cells in six SHIV-Ag85B-inoculated macaques (#501006, #508096, #509106, #803032, #901007, and #912080) and three SHIV-NI-inoculated macaques (#505060, #511128, and #711115) at 252 weeks after the SHIV89.6P challenge. After infusion, all of the macaques exhibited a depletion of CD8^+^ cells in peripheral blood (Fig. 10a). In the absence of CD8^+^ cells, two SHIV-Ag85B-inoculated macaques (#508096 and #912080) showed a rapid spike in plasma viral RNA, which subsequently declined to an undetectable level (Fig. 10b). On the other hand, plasma viral loads in all of the SHIV-NI-inoculated macaques were maintained at 10^4^ copies/ml of viral RNA after administration. One macaque (#511128) showed symptoms of AIDS at 2 weeks after administration. In contrast, in four SHIV-Ag85B-inoculated macaques (#501006, #509106, #803032, and #901007), plasma viral loads were not detected at any time point after CD8^+^ cell depletion (Fig. 10b). To detect viral RNA of the challenge virus only, viral RNA loads were determined by quantitative reverse transcriptase polymerase chain reaction (RT-PCR) for the SHIV vpr-specific region. Viral RNAs of SHIV89.6P were also detectable in two SHIV-Ag85B-inoculated macaques (#508096 and #912080) and all of the SHIV-NI-inoculated macaques after CD8^+^ cell depletion (Fig. 10c). In four SHIV-Ag85B-inoculated macaques (#501006, #509106, #803032, and #901007), viral RNAs of SHIV89.6P were not detected after CD8^+^ cell depletion (Fig. 10c).

To examine virus clearance in the animals, proviral DNAs in various lymphoid tissues of SHIV-Ag85B-inoculated macaques were measured by ultrasensitive digital PCR for SHIV-specific SIV *gag* gene regions. All of the SHIV-Ag85B and SHIV-NI-inoculated macaques were euthanized and taken to necropsy from 264 weeks after the challenge, and proviral DNA analyses were performed on necropsy lymphoid tissue samples (Fig. 10d). Two SHIV-Ag85B-inoculated macaques (#508096 and #912080) and all of the SHIV-NI-inoculated macaques showed plasma viremia at necropsy, and most of the infected macaques showed proviral DNA loads in the lymphoid tissues examined. In the SHIV-Ag85B-inoculated macaques (#501006, #509106, #803032, and #901007), proviral DNA was not detected in any of the lymphoid tissues analyzed (Fig. 10d). To detect proviral DNA of SHIV89.6P only, proviral DNA in lymphoid tissues prepared at the euthanasia time point was examined by PCR for the SHIV vpr-specific region. Two SHIV-Ag85B-inoculated macaques (#508096 and #912080) and all of the SHIV-NI-inoculated macaques showed proviral DNA of SHIV89.6P in the lymphoid tissues examined (Supplementary Fig. 9).

## Discussion

In the present study, we examined immune responses in cynomolgus macaques inoculated with an adjuvant molecule-expressing virus, SHIV-Ag85B, and also the SHIV-specific immunity induced by SHIV-Ag85B in the macaques after challenge with pathogenic SHIV89.6P. For this purpose, we genetically engineered a *nef*-deleted attenuated SHIV to harbor the *Ag85B* gene, so that Ag85B is produced locally when viral replication occurs. The SHIV-Ag85B-inoculated macaques showed a larger number of Gag/pol-specific IFN-γ-producing cells than that in SHIV-NI-inoculated macaques at 2 weeks after inoculation. Similarly, the SHIV-Ag85B-inoculated macaques showed a larger percentage of antigen-specific CD8^+^ T cells than that in the SHIV-NI-inoculated macaques. Our results suggest that the co-expression of a viral antigen and Ag85B stimulates antigen-specific cellular immune responses, which may help to increase the antiviral immune responses.

When we used cell lines, the replication of SHIV-Ag85B and that of parental SHIV-NI were similar. However, in the animal study, the viral loads in SHIV-Ag85B-inoculated macaques were lower than those in SHIV-NI-inoculated macaques. Moreover, *gag* DNAs in PBMCs of SHIV-Ag85B-inoculated macaques were not detected at the early stage of infection. Similar results were obtained in the second experiment. In contrast, *gag* DNAs in PBMCs of SHIV-NI-inoculated macaques were detected during the observation period. Our study also showed that inoculation of SHIV-Ag85B induced a remarkable antigen-specific T cell response against acute infection. One possible explanation is that the combination of Ag85B and a viral antigen mainly induced cellular immunity, as indicated by antigen-specific T cell responses, resulting in a more efficient immunomodulating effect against SHIV-Ag85B. On the other hand, it remains possible that the insertion of any gene into the Nef region may change the virulence of the virus in vivo or that the effect of Ag85B is non-specific. A better control virus would be insertion of an unrelated gene in the Nef region. Also, a previous study showed that Ag85B enhanced cellular immune responses. In a mouse model, co-injection of Ag85B plasmids with an HIV Env DNA vaccine enhanced antigen-specific cellular immune responses^18^. Thus, it might be possible to increase cellular immunity by co-injection of SHIV-NI together with Ag85B protein.

It is known that virus-specific cytotoxic T lymphocytes induced by an attenuated virus can provide protective immunity to a pathogenic virus. It has been reported that live attenuated SIV or SHIV vaccines can protect macaques against pathogenic SIV or SHIV challenges^8–10,26–28^. The expression of an inserted cytokine or chemokine gene from a genetically engineered vaccine virus provides adjuvant effects locally at the site of virus replication. Several studies have demonstrated that insertion of a cytokine in a gene-deleted live attenuated SIV could boost the immunogenicity of the virus and enhance its protection ability^17,29,30^. Vaccination of macaques with SIV expressing IFN-γ resulted in decreased viral loads and increased resistance to the challenge compared to vaccination with *nef*-deleted SIV, though it could not prevent persistent infection with a virulent challenge strain^30^. In this study, all of the macaques inoculated with SHIV-Ag85B did not show any plasma viral load and proviral DNA at 8 weeks after injection of the virus. Most of the SHIV-Ag85B-inoculated macaques, including those in the second experiment, did not show any evidence of SHIV89.6P infection at 20 weeks after the challenge with pathogenic SHIV89.6P (Fig. 4). Moreover, viral RNA and provirus DNA were not detected in the four SHIV-Ag85B-inoculated animals in adoptive transfer and CD8^+^ cell-depletion experiments (Figs. 9 and 10). It should be mentioned here that PBMCs isolated from SHIV-Ag85B-inoculated macaques showed robust IFN-γ ELISPOT responses and increases in the percentages of Gag/pol-specific monofunctional or polyfunctional CD8^+^ T cells in the acute phase of the pathogenic SHIV challenge. In contrast, protective immunity was not effectively induced in macaques in the group that received SHIV-NI. These results showing that the control against the highly pathogenic SHIV89.6P challenge afforded by SHIV-Ag85B suggest an alternative mechanism for a clinically useful prophylactic HIV/AIDS vaccine. However, in this study, we used SHIV89.6P as the challenge virus. Protection against CXCR4 tropic viruses such as SHIV89.6P is easier than that against CCR5 tropic viruses. Therefore, further studies are necessary to determine whether SHIV-Ag85B protects against the CCR5 tropic SIVmac239.

The live attenuated SIV or SHIV vaccines could induce not only cellular immune responses but also humoral immune responses^8–13^. Both SHIV-Ag85B and SHIV-NI elicited antigen-specific antibody responses in macaques. After the pathogenic SHIV89.6P challenge, neutralizing antibody titers in SHIV-NI-inoculated macaques were higher than those in SHIV-Ag85B-inoculated macaques at 12–24 weeks post challenge. Previous studies have shown that Ag85B enhanced cellular-mediated immunity, but the effect of Ag85B on humoral immune responses is unknown. In this study, despite inducing a higher neutralizing antibody titer against SHIV89.6P, SHIV-NI could not completely prevent pathogenic SHIV acquisition in macaques. These results showed that the neutralizing antibody response alone was not sufficient to control pathogenic SHIV.

Innate immune responses have been thought to play an important role in both the control and pathogenesis of HIV or SIV infection^31–34^. Furthermore, for the generation of adaptive immune responses, induction of innate immunity is crucial for vaccines to elicit potent antigen-specific immune responses. Attempts have been made to use various types of adjuvants for enhancing an immune response to an antigen, including vaccines or a therapeutic cure against HIV. In fact, poxvirus- and adenovirus-based vaccines required the addition of an adjuvant to induce effective immune responses^6,35,36^. For the generation of adaptive immune responses, induction of innate immunity is crucial for vaccines to elicit potent Ag-specific immune responses. As a result, it was demonstrated that SHIV-Ag85B might have a potent adjuvant activity as dsRNA recognized by the RIG-I receptor and it enhanced not only local innate immunity but also systemic adaptive immunity.

Indian rhesus macaque AIDS models possessing major histocompatibility complex (MHC)-I alleles, such as Mamu-A*01, Mamu-B*08, and Mamu-B*17, tend to show spontaneous viral control after an SIVmac challenge^37–40^. Therefore, analysis of the MHC allele is important for HIV vaccine research using AIDS macaque models. In a previous study, we established an AIDS model using cynomolgus macaques of Asian origin that exhibited MHC genetic diversity populations. Moreover, these MHC-I alleles showed more genetic diversity than those of Mauritian cynomolgus macaques^23,41^. We analyzed the MHC class I alleles of the macaques used in this study. As shown in Supplemental Table 1, the macaques had variable MHC class I alleles and we did not find restrictive MHC class I alleles in the animals used in this study that are similar to Mamu-A*01, Mamu-B*08, and Mamu-B*17 in rhesus macaques. We excluded macaques with MHC alleles that are known to confer enhanced SHIV control. We then used a stringent animal model involving a pathogenic SHIV.

Approximately 50% of the macaques vaccinated with a cytomegalovirus-based SIV vaccine manifested complete control of viral replication shortly after SIVmac239 infection^42–44^. Recently, it has been reported that the “Miami Monkey” achieved durable HIV remission through adeno-associated virus-based delivery of broadly neutralizing antibodies against SHIV^45^. In this study, the pathogenic virus was not detected in 57% of the macaques that had been injected with SHIV-Ag85B. Those macaques showed no detectable pathogenic virus measured as viral DNA in PBMCs and tissues or by adoptive transfer experiments and subsequent depletion of CD8^+^ cells.

Adoptive transfer and CD8 depletion studies are useful for assessing residual replication-competent AIDS virus^44,46,47^. The four SHIV-Ag85B-inoculated animals did not show viral RNA and provirus DNA in adoptive transfer and CD8 cell-depletion experiments. In previous studies, some live attenuated SIV vaccines were found to confer protection against an SIVmac challenge. However, levels of protection were variable. Also, those attenuated vaccines could not completely protect against or eliminate SIV^13,14,26,44^. Therefore, we could not exclude the possibility that exceedingly low levels of replication-competent virus may still exist in the macaques. However, there was a clear difference between the macaques that exhibited viral replication and those that did not in adoptive transfer and CD8^+^ cell-depletion experiments.

In summary, our results showed that immunization with SHIV-Ag85B provided protection from pathogenic infection and may have led to eradication of the challenge virus. In SHIV-Ag85B-inoculated macaques, SHIV antigen-specific CD8^+^ T cell responses with polyfunctionality were rapidly induced. Surprisingly, four of the SHIV-Ag85B-inoculated macaques did not show virus replication in an adoptive transfer experiment and a CD8^+^ cell-depletion study. These results suggest that SHIV-Ag85B elicited viral antigen-specific CD8^+^ T cell responses against pathogenic SHIV and provide the possibility of eradicating a pathogenic lentivirus from infected cells. The results of this study provide further insights into the containment of HIV infections as well as new opportunities to develop a better therapeutic cure and vaccines.

## Materials and methods

### Construction of SHIV-Ag85B

SHIV-NM3rN, having HIV-1 *NL432* genes on an SIVmac239 background, was utilized as a starting material. A recombinant SHIV was constructed according to the method reported previously^15,16^. The SHIV-nef vector, designated as SHIV-NI, was constructed from an infectious molecular clone of SHIV-NM3rN^48^. The source of the *env* gene of SHIV-NI was the X4-tropic virus HIV-1 NL432. In SHIV-NI, the *nef* gene was replaced by some unique restriction enzyme sites, including ClaI and ApaI sites. The *Ag85B* gene was amplified by PCR from *Mycobacterium kansasii* as a template using 5′-ATATCGATACCATGTTCTCCCGTCCCGGGCT-3′ (ClaI) and 5′-TAGGGCCCCTAGCGGGCGCCCAGGCTGG-3′ (ApaI) primers. The PCR product was then digested with restriction enzymes for ClaI and ApaI sites. This plasmid was designated as pSHIV-Ag85B. SHIV-Ag85B was prepared by transfecting pSHIV-Ag85B into 293T cells using FuGENE 6 Transfection Reagent (Roche Diagnostics, Indianapolis, IN), and the culture supernatant was stored at 48 h after transfection in liquid nitrogen until use.

### Virus stocks

SHIV-Ag85B, SHIV-NI, and SHIV89.6P were used in this study. These viral stocks were propagated in PBMCs from cynomolgus macaques. Briefly, PBMCs were separated by a standard Ficoll density gradient separation method and cultured in RPMI1640 supplemented with 10% fetal bovine serum, 2 mM L-glutamine, and 100 units/ml of IL-2 (Shionogi) and then stimulated with phytohemagglutinin for 72 h. The cells were infected with SHIV-Ag85B, SHIV-NI, or SHIV89.6P at a multiplicity of infection (MOI) of 0.1. Half of the culture medium was replaced with a fresh culture medium every 3 days, and cell-free supernatants were collected between 6 and 9 days after infection. The TCID_50_ of each of the SHIVs was measured using M8166 cells. The TCID_50_ values of viral stocks were 5 × 10^4^ for SHIV-Ag85B, 4.7 × 10^4^ for SHIV-NI, and 3 × 10^5^ for SHIV89.6P.

### Detection of Ag85B protein

M8166 cells were infected with SHIV-Ag85B at an MOI of 0.1 and incubated for 1 h. The cells were washed three times with phosphate-buffered saline (PBS) and then incubated for another 48 h in the culture medium. After three further washings with PBS, the cells were lysed in PBS containing 1.5 M urea, 2% NP-40, and 5% 2-mercaptoethanol and then separated by sodium dodecyl sulfate-polyacrylamide gel electrophoresis, transferred by electroblotting onto a nitrocellulose membrane, and blocked with 5% nonfat dry milk in PBS containing 0.01% Tween 20 (PBST). Following three washings with PBST, the membrane was incubated with a rabbit anti-Ag85B polyclonal antibody for 2 h. The membrane was washed three times with NBT/BCIP (Roche Diagnostics, Mannheim, Germany) before being incubated with alkaline phosphatase-labeled anti-rabbit IgG (New England Biolabs, Beverly, MA).

### Virus replication in macaque cells and human cells

To investigate the kinetics of virus replication, cynomolgus macaque PBMCs and CEM×174 cells were infected with SHIV-Ag85B or SHIV-NI at an MOI of 1 and incubated for 1 h. Half of the culture supernatant was harvested with subsequent addition of a new medium every 3 days. Virus replication kinetics was monitored by SIV Gag p27 production in the supernatant using an SIV core antigen ELISA assay kit (Beckman Coulter, Miami, FL).

### Innate immune responses

We used cynomolgus macaque PBMCs prepared from three animals in this experiment. Cynomolgus macaque PBMCs (2 × 10^5^ cells per well) and CEM×174 cells (2 × 10^5^ cells per well) were infected with SHIV-Ag85B or SHIV-NI at an MOI of 0.2 and cultured for 48 h. The cells were harvested, and mRNA was extracted using an RNeasy Mini Kit (QIAGEN) and then reverse-transcribed to cDNAs using an Omuniscript system (QIAGEN). The cDNA was subjected to real-time PCR for IFN-α, IFN-β, IFN-γ, TNFα, RIG-I, MDA5, and β-actin selectin using a LightCycler (Roche Applied Science, Tokyo, Japan). The specific primers for each target (listed below) and probes were designed by Universal ProbeLibrary Assay Design Center (Roche Applied Science). Primers used in this study were 5′-CCCTCTCTTTATCAACAAACTTGC-3′ and 5′-TTGTTTTCATGTTGGACCAGA-3′ for IFN-α, 5′-CGACACTGTTCGTGTTGTCA-3′ and 5′-GAAGCACAACAGGAGGAGCAA-3′ for IFN-β, 5′-GGCATTTTGAAGAATTGGAAAG-3′ and 5′-TTTGGATGCTCTGGTCATCTT-3′ for IFN-γ, 5′-AGCCCATGTTGTAGCAAACC-3′ and 5′-TCTCAGCTCCACGCCATT-3′ for TNFα, 5′-TGGACCCTACCTACATCCTGA-3′ and 5′-GGCCCTTGTTGTTTTTCTCA-3′ for RIG-I, 5′-AGGCACCATGGGAAGTGAT-3′ and 5′-GGTAAGGCCTGAGCTGGAG-3′ for MDA5, and 5′-CCAACCGCGAGAAGATGA-3′ and 5′-CCAGAGGCGTACAGGGATAG-3′ for β-actin.

### Ethical statement

The animals were housed in animal biosafety level 3 facilities at Tsukuba Primate Research Center (TPRC) of National Institutes of Biomedical Innovation, Health and Nutrition (NIBIOHN). The studies were performed in TPRC, NIBIOHN after approval by the Committee on the Ethics of Animal Experiments of NIBIOHN in accordance with the guidelines for animal experiments at NIBIOHN. The animals were used under the supervision of the veterinarians in charge of the animal facility. The endpoint for euthanasia was determined by typical clinical symptoms of AIDS, such as weight loss, diarrhea, and neurologic syndrome. These diagnostics were performed by the veterinarians.

### Animals

We used 38 adult cynomolgus macaques (from Indonesia, Philippines, and Malaysia), all of which were negative for SIV, simian type D retrovirus, simian T cell lymphotropic virus, simian foamy virus, Epstein–Barr virus, cytomegalovirus, and B virus. Seven cynomolgus macaques were intravenously inoculated with 10^4^ TCID_50_ of SHIV-Ag85B, five macaques were intravenously inoculated with 10^4^ TCID_50_ of SHIV-NI, and another five macaques were intravenously inoculated with 10^4^ TCID_50_ of SHIV89.6P (first experiment). To evaluate viral replication, a further seven macaques were inoculated intravenously with SHIV-Ag85B (second experiment; Exp 2). To evaluate the protective efficacy of SHIV-Ag85B or SHIV-NI in inoculated macaques, the macaques were challenged intravenously with pathogenic SHIV89.6P at 37 weeks after inoculation. As controls, a further five naive macaques were challenged intravenously with SHIV89.6P. Blood was collected periodically using sodium citrate as an anticoagulant and used for determination of CD4^+^ T cell counts, quantification of plasma viral loads, and immunological analysis. Lymphoid tissue samples were obtained by biopsy at 34 weeks after virus infection and were used for determination of proviral DNA loads and histopathology. For the adoptive transfer experiment, 15 ml of blood and 1–3 × 10^7^ cells of lymphoid tissues from SHIV-Ag85B- or SHIV-NI-inoculated macaques, collected at 212 weeks after the SHIV89.6P challenge, were infused intravenously into healthy macaques (experiment 3). In the third experiment, nine naive macaques were used as SHIV-naive recipients. Viral and proviral DNA loads were assessed in recipient macaques during the observation period after adoptive transfer. For the CD8^+^ cell-depletion experiment, macaques received a single intravenous infusion of 10 mg/kg of a CD8α CDR-grafted rhesus IgG1 antibody (M-T807) (NIH Nonhuman Primate Reagent Resource). Antibody cTM-T807 was intravenously inoculated at 252 weeks after the challenge. CD8^+^ T cell counts and viral and proviral DNA loads were assessed in recipient macaques during the observation period after the CD8^+^ depletion experiment. The design of the macaque study is outlined in Fig. 2a.

### Preparation of DNA samples and amplification of the SHIV *gag* gene by nested PCR

For determining the proviral DNAs in SHIV-Ag85B-inoculated macaques, nested PCR was used to amplify a fragment of the *gag* gene segment. Proviral DNA was extracted from PBMCs of the inoculated macaques. Cellular DNAs were extracted using DNeasy tissue kits (QIAGEN). Nested PCR was performed using TaKaRa Ex Taq (Takara Bio Inc., Shiga, Japan). The initial and nested PCR protocols have been described elsewhere^49,50^. Primers used in this study were Outer SIV*gag*-F (5’-CCATTAGTGCCAACAGGCTCAG-3’) and Outer SIV*gag*-R (5’-CCCCAGTTGGATCCATCTCCTG-3’) for first-round PCR and Nested SIV*gag*-F (5’-ACTGTCTGCGTCATCTGGTG-3’) and Nested SIV*gag*–R (5’-GTCCCAATCTGCAGCCTCCTC-3’) for second-round PCR. After the second amplification, 10 μl of the nested PCR-amplified product was run on 1.0% agarose gel, and DNA bands were visualized by staining with ethidium bromide. The lowest concentration of plasmid SIV DNA that could be detected with this PCR method in the first amplification with the outer gag primer pair was 100 copies. Upon further amplification with nested/internal gag primers, a single copy of plasmid DNA could be routinely detected^49,50^.

### Stability of the inserted *Ag85B* gene in vivo

Proviral DNA was extracted from 1 × 10^6^ PBMCs of the inoculated macaques. When the virus was re-isolated, CD8^+^-depleted PBMCs, which were co-cultured with M8166 cells, were also monitored. Cellular DNAs were extracted using DNeasy tissue kits (QIAGEN). To check the stability of the inserted *Ag85B* gene in SHIV-Ag85B, the proviral DNA fragments covering the inserted *Ag85B* gene in SHIV-Ag85B were amplified by PCR with primers. The primer sequences are shown above.

### Plasma viral RNA loads

The levels of SHIV infection were monitored by measuring plasma viral RNA loads using highly sensitive quantitative real-time RT-PCR as described previously^23,51,52^. Briefly, viral RNA was isolated from plasma using a MagNA PureCompact Nucleic Acid Isolation Kit (Roche Diagnostics). Real-time RT-PCR was performed using a QuantiTec Probe RT-PCR Kit (Qiagen) and a LightCycler 480 thermocycler (Roche Diagnostics, Rotkreuz, Switzerland). The SIVmac239 *gag* gene was amplified with the probe 5′-FAM-TGTCCACCTGCCATTAAGTCCCGA-TAMRA-3′ (where FAM is 6-carboxyfluorescein and TAMRA is 6-carboxytetramethylrhodamine) and the primers 5′-GCAGAGGAGGAAATTACCCAGTAC-3′ and 5′-CAATTTTACCCAGGCATTTAATGTT-3′. The limit of detection was calculated to be 100 viral RNA copies per ml. The SHIV89.6P *vpr* gene was amplified with the probe 5′-FAM-AGGACCACAAAGGGAACCATGGGATG-TAMRA-3′ and the primers 5′-TGGAAGAAAGACCTCCAGAAAATG-3′ and 5′-CAAGTGCAGTTAGCAAGCGAGGAT-3′. The limit of detection was calculated to be 1000 viral RNA copies per ml.

### Proviral DNA loads

A DNA sample was extracted from PBMCs and lymphoid tissues using a DNeasy tissue kit (QIAGEN) according to the manufacturer’s protocol. Ultrasensitive digital PCR was performed in the QX200 Droplet Digital PCR system (Bio-Rad). Twenty μl of a reaction mixture containing 2 μl of a DNA sample, ddPCR supermix for probes (no dUTP) (Bio-Rad), 900 nM of each primer, 200 nM of the probe, and demineralized water was prepared. The mixture was placed into the DG8 cartridge with 70 μl of droplet generation oil (Bio-Rad), and the droplets were formed in the droplet generator (Bio-Rad). Subsequently, the droplets were transferred to a 96-well microplate. PCR amplification was performed using the following program: initial denaturation and stabilization at 95 °C for 10 min, 40 cycles of denaturation at 94 °C for 30 s, and annealing/extension at 57 °C for 60 s, followed by 10 min at 98 °C. Subsequently, the droplets were sorted and analyzed in a QX200 droplet reader (Bio-Rad) using the software QuantaSoft v1.6 (Bio-Rad). Samples were only considered if >20,000 droplets were read. The cell numbers were monitored by highly sensitive quantitative real-time PCR as described previously^53^. DNA samples were extracted from PBMCs and lymphoid tissues using a DNeasy tissue kit (QIAGEN) according to the manufacturer’s protocol. The cell number was validated by detection of the cellular IL-4 sequence with the rhesus IL-4-specific primers 5′-TGTGCTCCGGCAGTTCTACA-3′ and 5′-CCGTTTCAGGAATCGGATCA-3′ and the probe 5′-FAM-TGCACAGCAGTTCCACAGGCACAAG-TAMRA-3′.

### Preparation of DNA samples and amplification of the SHIV *vpr* gene by nested PCR

To detect proviral DNA of the challenge virus only, nested PCR was used to amplify a fragment of the SHIV*vpr* gene segment. A DNA sample was extracted from PBMCs and lymphoid tissues using a DNeasy tissue kit (QIAGEN) according to the manufacturer’s protocol. Nested PCR was performed using TaKaRa Ex Taq (Takara Bio Inc., Shiga, Japan). The initial and nested PCR protocols have been described elsewhere^52^. Primers used in this study were Outer SHIV*vpr*-F (5’-ATCCCACCTGGAAACAGTGGAGAAGAGACA-3’) and Outer SHIV*vpr*-R (5’-TCTCCGCTTCTTCCTGCCATAGGAGATG-3’) for first-round PCR and Nested SHIV*vpr*-F (5’-CGGTAAACCACCTACCAAGGGAGCTAATTT-3’) and Nested SHIV*vpr*–R (5’-CAAGCAGTTTTAGGCTGACTTCCTGGATGC-3’) for second-round PCR.

### CD4^+^ and CD8^+^ T cell counts

One hundred microliters of whole blood from each of the cynomolgus macaques was stained with combinations of fluorescence-conjugated monoclonal antibodies: anti-CD3 (clone SP34-2, Alexa700; BD), anti-CD4 (clone L200, PerCP-Cy5.5; BD), and anti-CD8 (clone DK25, APC; Dako). Flow cytometry was performed on a FACSCanto II flow cytometer (BD). The data were analyzed using the FACSDiVa software.

### Immunohistochemistry

Lymphoid tissue samples were fixed in 4% paraformaldehyde in PBS at 4 °C overnight and embedded in paraffin wax. Sections were deparaffinized by pretreatment with 0.5% H_2_O_2_ in methanol and then subjected to antigen retrieval with target retrieval solution (Dako S1700, pH 6.1) followed by heating in an autoclave for 20 min at 121 °C. The sections were then incubated with an anti-human CD4 mouse monoclonal antibody (1:30; NCL-CD4; Novocastra Laboratories Ltd., UK) or an anti-SIV mouse monoclonal antibody (1:100; SIV-Nef; Santa Cruz Biotechnology) at 4 °C for 24 h. Following brief washes with a buffer, the sections were incubated with the EnVision^TM^+ Dual Link-HRP system (Dako) as a secondary stage for 60 min. Labeling was “visualized” by treating the sections with chromogen 3,3’-diaminobenzidine tetroxide (Dojin Kagaku, Japan) and H_2_O_2_. The sections were then counterstained with hematoxylin.

### HIV Env-specific antibody titer

HIV Env-specific antibody responses were determined as previously described^54^. Levels of SHIV Env-specific antibodies in plasma were measured by an ELISA using purified gp160 protein. Briefly, ELISA plates were coated with 100 ng per well of soluble gp160 protein. Serially diluted plasma was assayed for each animal. Horseradish peroxidase (HRP)-conjugated anti-monkey IgG (NORDIC IMMUNOLOGY) was used for detection with TMB HRP substrate solution (Dako). The absorbance at 450 nm was read using a plate reader (Thermo). Assays were performed in duplicate in each experiment.

### Neutralization assay

Neutralization was measured as the ability of plasma samples to reduce virus infection of TZM-bl cells as previously described^55,56^. Briefly, heat-activated plasma was incubated with SHIV89.6P for 60 min at 37 °C. TZM-bl cells were added and allowed to incubate for 48 h. A luciferase reagent (Britelite™; PerkinElmer, Boston, MA) was added, and luminescence was measured. Results were reported as the 50% inhibitory dilution, which is the dilution of serum resulting in 50% reduction in luminescence compared to that of virus control wells.

### Analysis of polyfunctional Gag/pol-specific T cell responses

Antigen-specific cellular immune responses were assessed in multiparameter ICS assays as described previously^57,58^. Briefly, thawed cryopreserved PBMCs were co-cultured for 6 h with autologous herpes virus papio-immortalized B-LCL cells that had been infected with a recombinant vaccinia virus expressing SIV Gag/pol. They were incubated with brefeldin A (BD) for the final 5 h of stimulation. Then immunostaining was performed using a CytofixCytoperm kit (BD) and the following monoclonal antibodies: anti-CD3 (clone SP34-2, Alexa700; BD), anti-CD4 (clone L200, APC-H7; BD), anti-CD8 (clone DK25, APC; Dako), anti-IFN-γ (clone 4 S.BS, PE; BD), anti-TNF-α (clone MAM11 PE-Cy7), and anti-IL-2 (clone MQ-1-17H12). A fixable-dead-cells stain kit (Invitrogen) was used to exclude dead cells from the analysis. Samples were fixed with 1% freshly prepared paraformaldehyde for at least 1 h and then analyzed in a FACSCanto II flow cytometer (BD). Data were analyzed using FACSDiVa (BD) software.

### Antigen-specific IFN-γ ELISPOT assay

The number of antigen-specific IFN-γ-producing cells in PBMCs was determined by ELISPOT analysis as described previously^23^. Ninety-six-well, flat-bottom plates were coated with an anti-IFN-**γ** monoclonal antibody (clone MD-1; U-Cytech, Utrecht, Netherlands) and blocked with 2% bovine serum albumin in PBS. Briefly, thawed cryopreserved PBMCs were co-cultured for 9 h with autologous herpes virus papio-immortalized B-LCL cells that had been infected with a recombinant vaccinia virus expressing SIV Gag/pol or Ag85B and parental vaccinia virus (pVV) as a control. Gold-labeled anti-biotin IgG solution (U-Cytech, Utrecht, Netherlands) was added to the washed plates and the plates were then incubated for 1 h at 37 °C. Spot-forming cells (SFCs) were counted using the KS ELISPOT compact system (Zeiss) after a 15-min reaction with an activator mix (U-Cytech, Utrecht, Netherlands). An SFC was defined as a large black spot with a fuzzy border. After subtraction of SFCs attributable to pVV, a positive ELISPOT response was defined as a level of SFC/10^6^ PBMCs at least three times the mean SFCs of the pre-experiment macaques and 50 SFCs over the mean pre-experiment macaque values.

### Memory phenotype of SHIV-specific CD8^+^ T cells

PBMCs treated as described above were immunostained using a CytofixCytoperm kit (BD) and the following monoclonal antibodies: anti-CD3 (clone SP34-2, Alexa700; BD), anti-CD4 (clone L200, APC-H7; BD), CD8 (clone DK25, APC; Dako), CD28 (clone CD28.2, ECD; Beckman-Coulter), CD95 (clone DX2, PE-Cy7; BD), and IFN-γ (clone 4 S.BS, PE; BD). A fixable-dead-cells stain kit (Invitrogen) was used to exclude dead cells from the analysis. The percentages of CD8^+^ T cells in CM and EM populations were determined by using a combination of CD28 and CD95 markers. Samples were fixed with 1% of freshly prepared paraformaldehyde for at least 1 h and then analyzed using a FACSCanto II flow cytometer (BD). The data analysis was conducted using the FACSDiVa (BD) software.

### Statistical analysis

Statistical analyses and AUC analysis were performed using the Prism software (GraphPad Software, Inc.) with significance set at a *P* value of <0.05. Comparisons were performed by Wilcoxon signed-rank test, and multiple comparisons were performed by Kruskal–Wallis test and two-way analysis of variance with Tukey’s test. Correlation analyses were performed by Spearman’s test.

### Reporting summary

Further information on research design is available in the Nature Research Reporting Summary linked to this article.

## Supplementary information

Supplementary Information

Reporting Summary

## Data Availability

All data generated in this study are available from the corresponding author upon reasonable request.
