# Supplementary material for: Long-term protective immunity induced by an adjuvant-containing live-attenuated AIDS virus
Source: NPJ Vaccines. 2021 Oct 22;6:124. doi: 10.1038/s41541-021-00386-5 (PMC8536741; doi:10.1038/s41541-021-00386-5)
Supplement: Supplementary file 1 — Supplementary Information [file 41541_2021_386_MOESM1_ESM.pdf]

Supplementary Table 1. MHC of cynomolgus macaques used in this study

Macaque	MHC class I *Mafa-A*	MHC class I *Mafa-B*
#501006	*A1*043:12, A1*078:01:01, A2*05:57:01*	*B*019:04, B*028:06, B*046:10:02, B*060:01:01:01, B*117:02:01, B*117:05, B*124:01:01, B*140:01:02, B11L*01:04*
#509108	*A1*018:03, A1*069:01, A1*103:01, A2*05:06:02*	*B*052:02, B*058:02, B*089:01:05, B11L*01:04*
#508096	*A1*010:02:01, A3*13:15*	ND
#509106	*A1*089:01, A2*05:16*	*B*064:01:01:01, B*081:02, B*083:01, B*101:07, B*104:01:02:01*
#803032	*A1*059:02, A1*060:06:02, A1*097:01, AG*06:01*	*B*013:09, B*060:03:01, B*063:01, B*137:03:01:01*
#901007	*A1*032:01, A1*066:06:01:01,*	*B*060:03:01, B*077:03, B*090:01, B*151:01*
#912080	*A1*068:03*	*B*004:03, B*007:01:01, B*011:01, B*121:01*
#505060	*A1*055:03, A1*064:03, A1*067:05, A1*067:06*	*B*007:01:05:02, B*022:01:02, B*060:13:01:02, B*069:02, B*069:08, B*174:01:01:01N*
#511128	*A1*010:02:01*	*B*004:03:01, B*019:04, B*140:01:02*
#510113	*A1*003:01, A1*010:02:01*	*B*064:01:01:01*
#711115	*A1*067:01:01:01, A1*067:06, A1*078:01:01, A1*078:01:02*	*B*007:01:01:02, B*060:03:01, B*137:03:01:01, B*203:02, B11L*01:04*
#801008	*A1*023:02, A1*089:03*	*B*021:01, B*028:03:01:01, B*036:04, B*099:01, B*108:01, B*124:01:01*

#073	*A1*036:02, A1*043:05, A1*067:01:01:01, A1*067:06, A1*089:01*	*B*013:20, B*057:02:01:01, B*104:01:02:01, B*134:02:01:01*
#075	*A1*003:02, A2*24:09, A3*13:29*	*B*007:01:01:02, B*007:01:05:02, B*007:11, B*021:05, B*057:03:01:01, B*085:01:01:01, B*098:04:01:01, B*118:01*
#076	*A1*010:02:01, A1*010:04, A1*010:08, A1*043:14*	*B*013:09, B*045:02, B*060:03:01, B*060:13:01:02, B*090:03, B*137:03:01:01*
#077	*A1*010:02:01,*	*B*011:04, B*011:05:02, B*060:02, B*060:03:01, B*060:13:01:02, B*075:01:01:01, B*203:02*
#078	*A1*022:05, A1*043:12, A1*089:02*	*B*041:02:01:03, B*045:07:01:01, B*048:03, B*151:02:01, B*159:01*
#079	*A1*009:01*	*B*002:03, B*069:05, B*069:07*
#080	*A1*009:01*	*B*069:05*

ND, not detected.

Supplementary Fig. 1

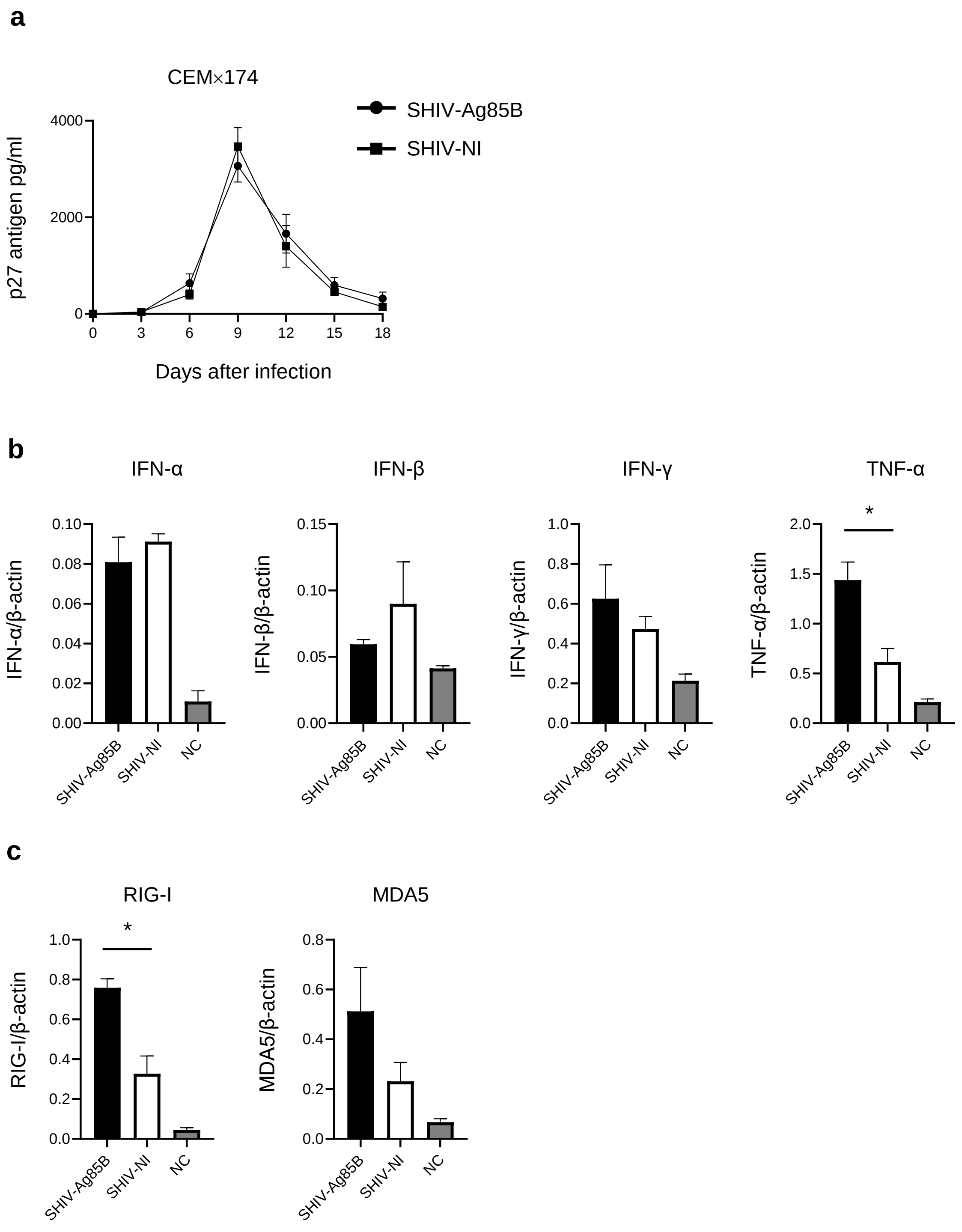

Supplementary Fig. 1 a Virus replication kinetics of SHIV-Ag85B and SHIV-NI in CEM×174 cells. Representative results of three independent experiments are shown. **b**, **c** CEM×174 cells were infected with SHIV-Ag85B and SHIV-NI for 48 h, and the increases in mRNA levels of IFN-α, IFN-β, IFN-γ, TNF-α, and RIG-I and MDA5 were determined by real-time PCR. Fold increase of each target gene was normalized to β-actin, and the expression levels are represented as relative values to the control. Control is uninfected cells. Data are averages of triplicate samples from three identical experiments and error bars represent means ± SEM. Statistical analysis were performed using the Kruskal-Wallis test. * $P < 0.05$.

Supplementary Fig. 2

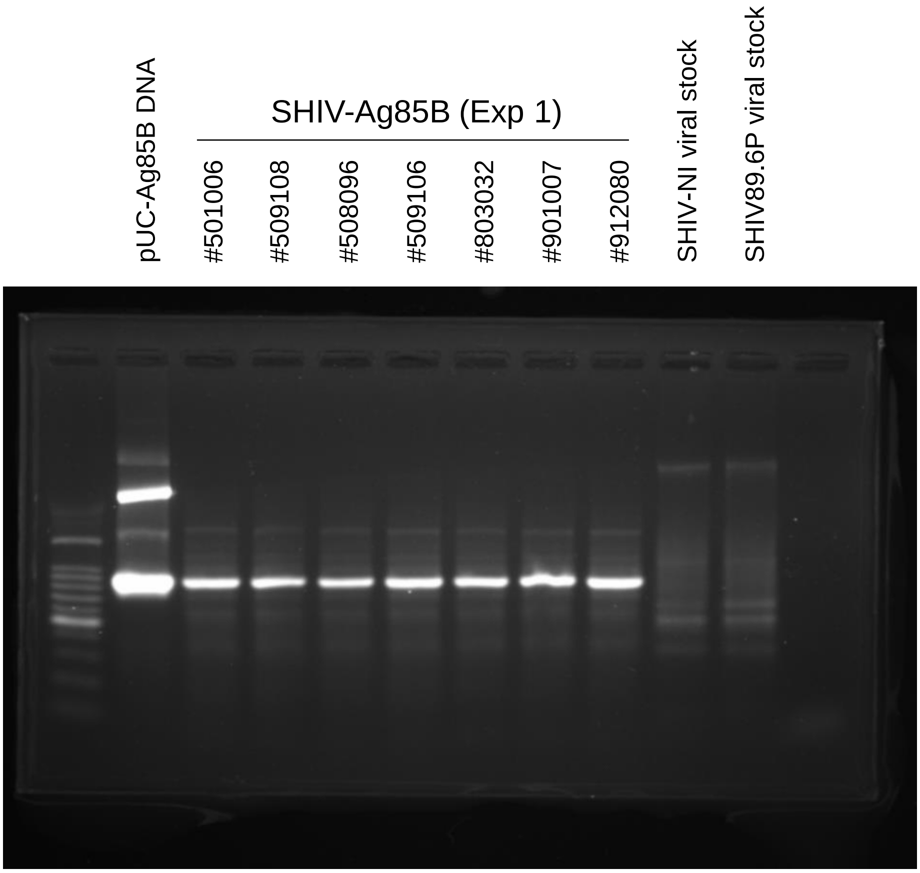

Supplementary Fig. 2 Stability of the *Ag85B* gene. Stability of the inserted *Ag85B* gene in SHIV-Ag85B (Exp 1). Proviral DNAs in PBMCs from macaques inoculated with SHIV-Ag85B were isolated, and the stability of the inserted *Ag85B* gene in SHIV-Ag85B was analyzed by PCR. DNA plasmids of Ag85B, SHIV-NI cDNA and SHIV89.6P cDNA were used as templates for controls.

Supplementary Fig. 3

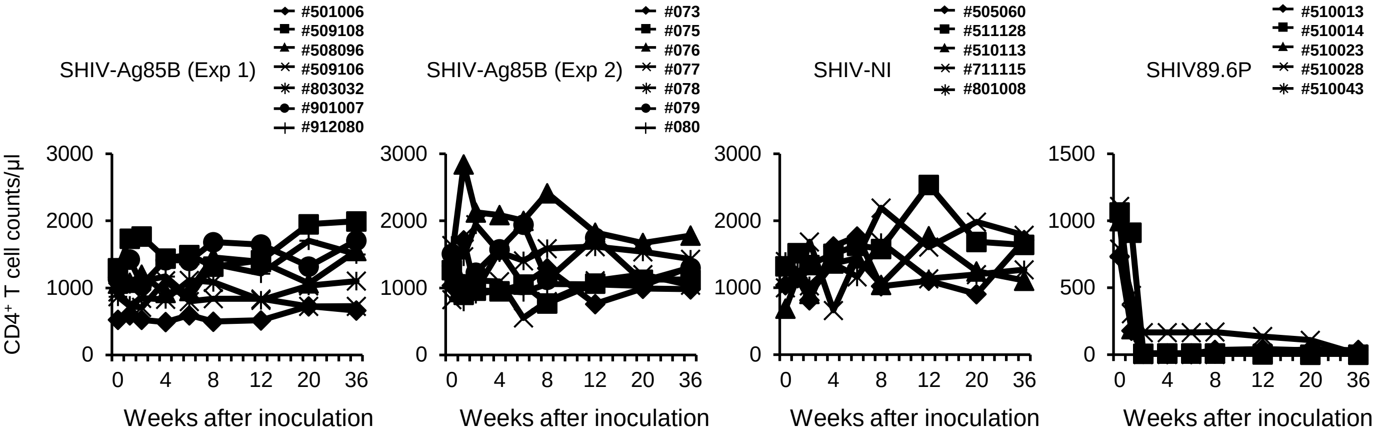

Supplementary Fig. 3 Count of CD4$^+$ T cells after SHIV-Ag85B (Exp 1), SHIV-Ag85B (Exp 2), SHIV-NI and SHIV89.6P infection in cynomolgus macaques. Whole blood was stained by CD3, CD4 and CD8 Abs, and CD4$^+$ T cell counts were determined by flow cytometric analysis.

Supplementary Fig. 4

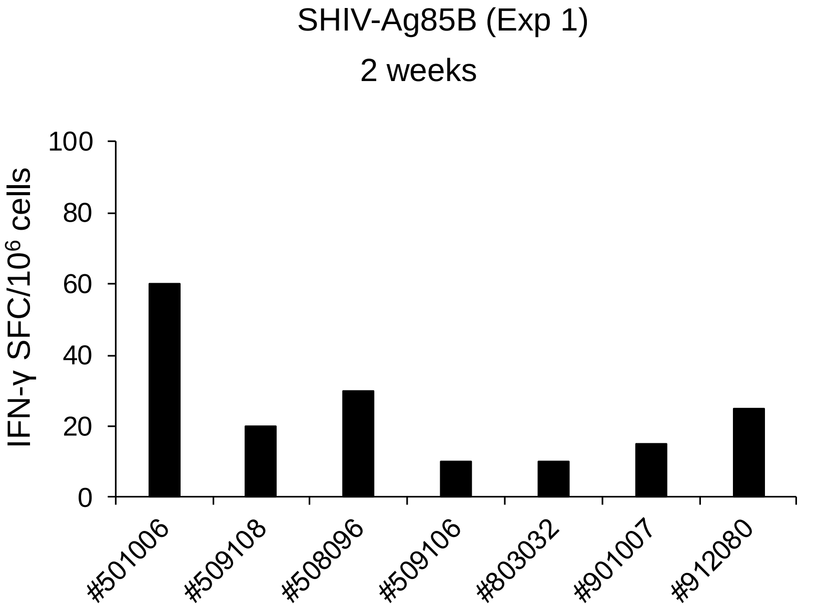

Supplementary Fig. 4 Ag85B-specific IFN-γ ELISPOT responses in macaques inoculated with SHIV-Ag85B. Number of Ag85B-specific IFN-γ-producing cells in macaque PBMCs after inoculation with SHIV-Ag85B (Exp 1). PBMCs obtained at 2 weeks after SHIV-Ag85B inoculation were co-cultured for 6 h with autologous B-LCL cells that had been infected with a recombinant vaccinia virus expressing Ag85B. Antigen-specific IFN-γ ELISPOT results are depicted as spots per 10^6 PBMCs.

Supplementary Fig. 5

a

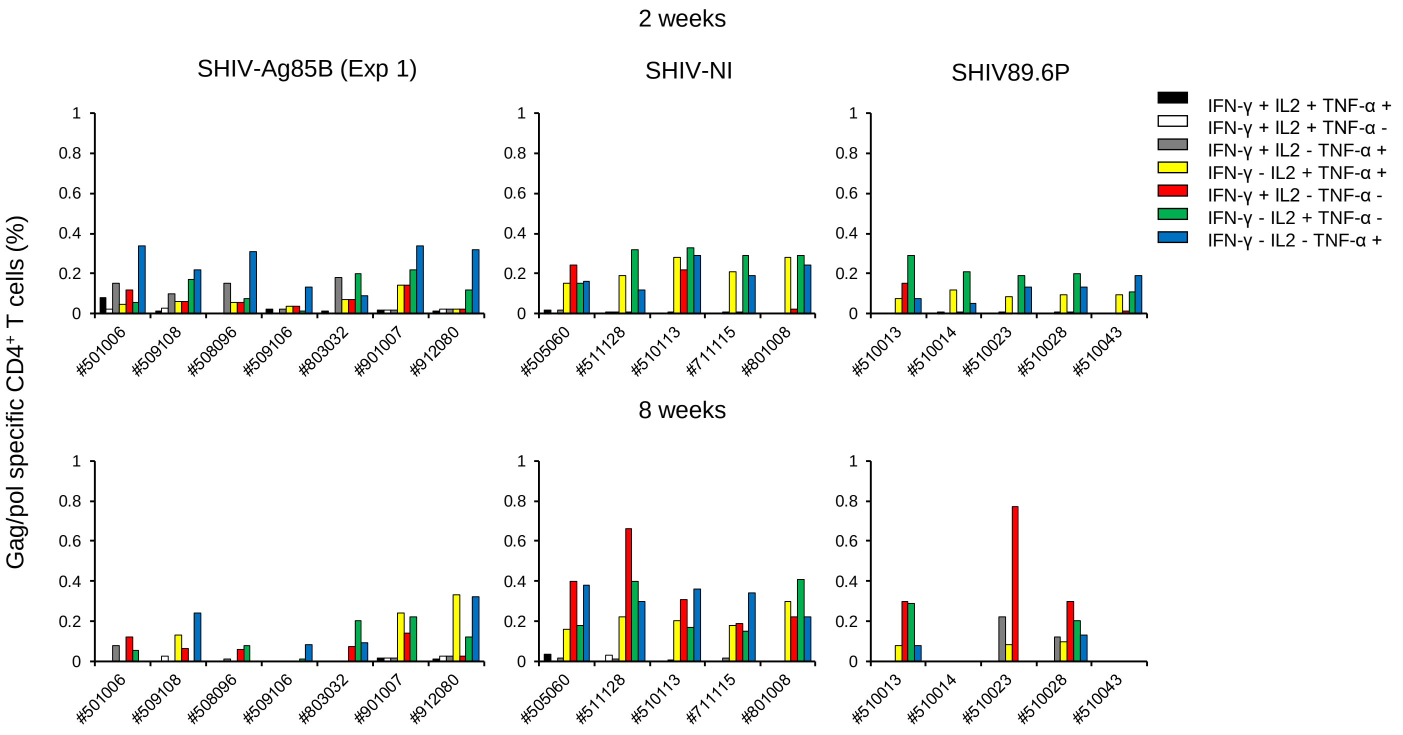

b

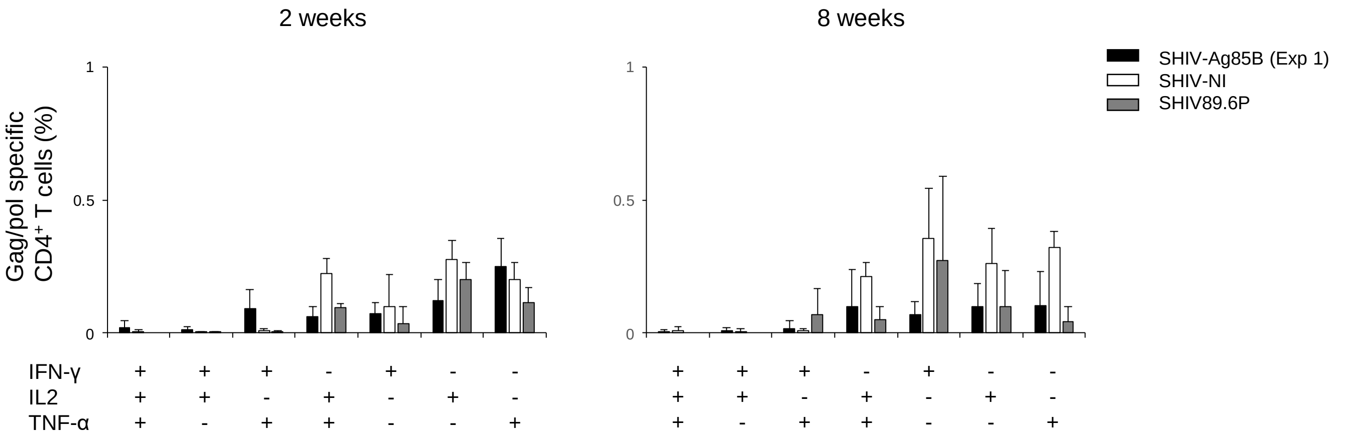

Supplementary Fig. 5 a SHIV antigen-specific $CD4^+$ T cell responses in macaques inoculated with SHIVs. Percentages of Gag/pol-specific $CD4^+$ T cells producing IFN-γ, TNF-α, and IL2 in macaques inoculated with SHIV-Ag85B (Exp 1), SHIV-NI and SHIV89.6P. The cytokine profile in cells was determined by flow cytometry by gating for lymphocytes and $CD4^+$ T cells. PBMCs obtained at 2 weeks after infection with SHIVs were co-cultured for 6 h with autologous B-LCL cells that had been infected with a recombinant vaccinia virus expressing SIV Gag/pol. **b** Mean percentages of Gag/pol-specific induction of single or multiple cytokines in SHIV-Ag85B (Exp 1)-inoculated macaques, SHIV-NI-inoculated macaques and control macaques after SHIV89.6P challenge. Error bars represent means ± SEM. Statistically significant differences between SHIV-Ag85B (Exp 1) and SHIV-NI were determined by using Student's *t* test.

Supplementary Fig. 6

a

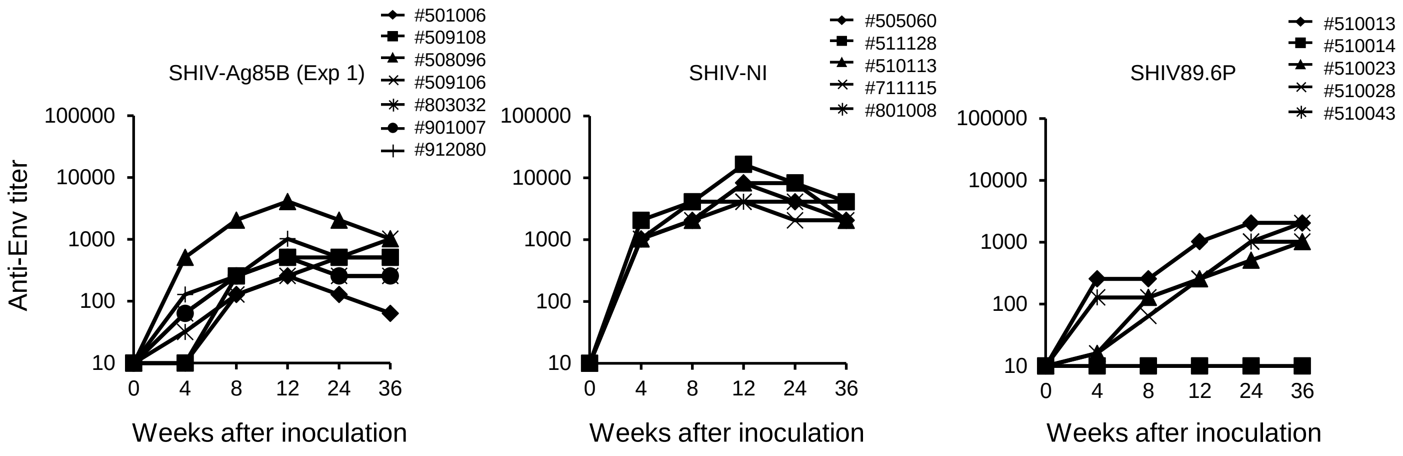

b

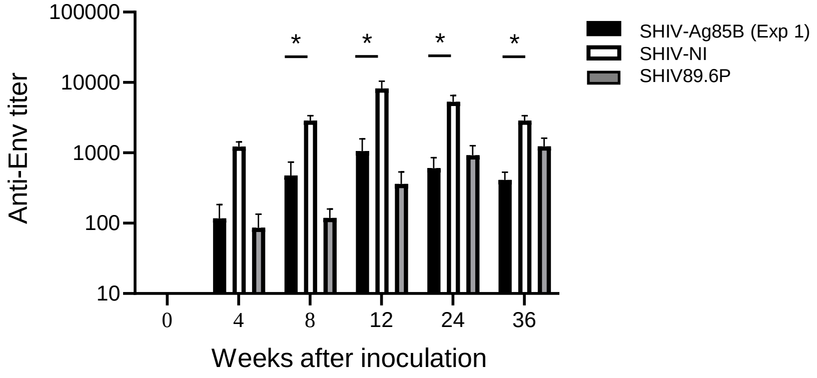

Supplementary Fig. 6 a HIV Env-specific antibody responses in macaques inoculated with SHIVs. The endpoint titers of plasma in SHIV-Ag85B (Exp 1), SHIV-NI, and SHIV89.6P-inoculated macaques were measured by ELISA. **b** Means of HIV Env-specific antibody titers in SHIV-Ag85B (Exp 1)-inoculated macaques, SHIV-NI-inoculated macaques and control macaques. Error bars represent means ± SEM. Statistical analysis was performed using two-way analysis of variance with Tukey's test * $P < 0.05$.

Supplementary Fig. 7

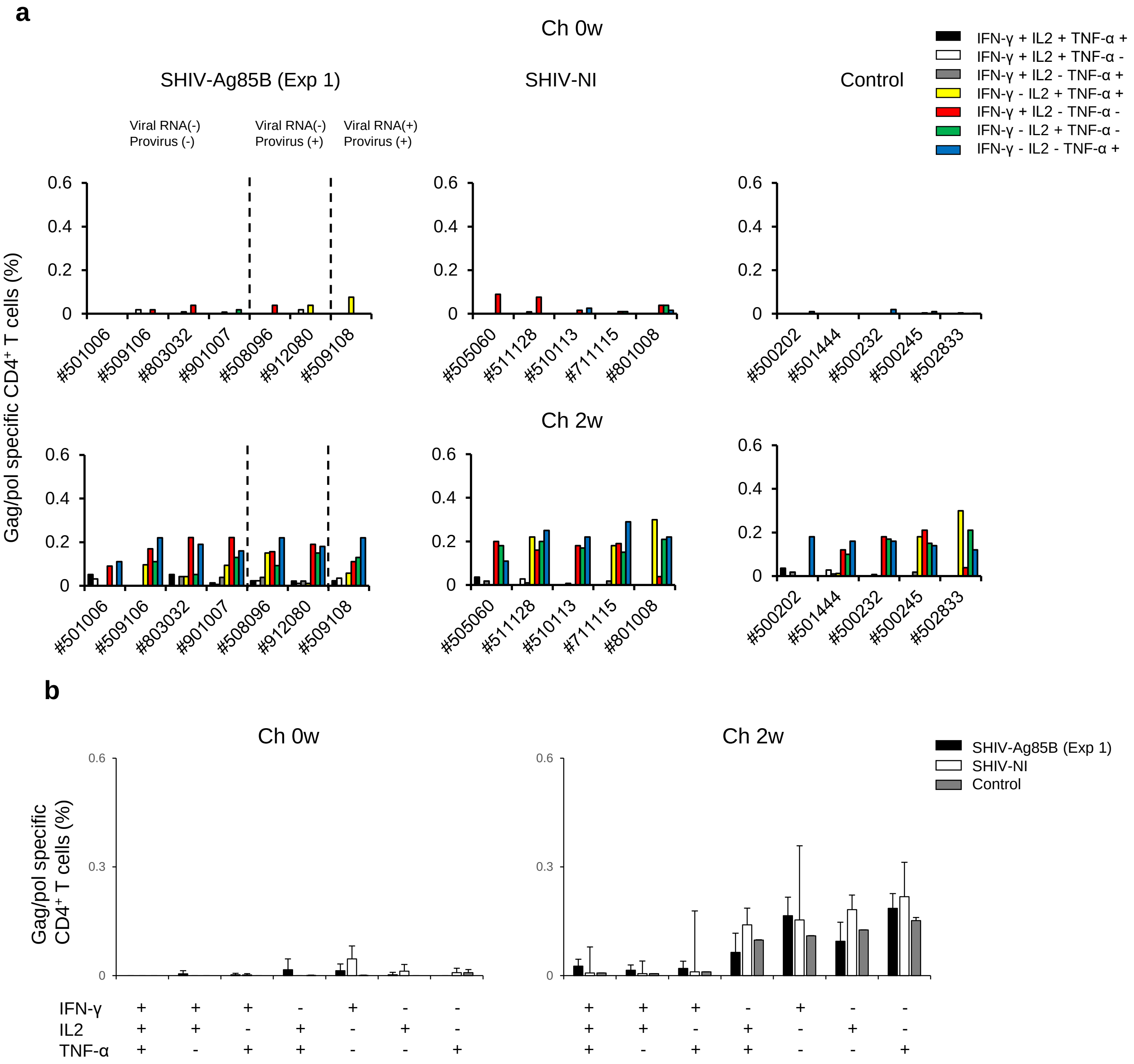

Supplementary Fig. 7 a SHIV antigen-specific $CD4^+$ T cell responses after pathogenic SHIV89.6P challenge. Percentages of Gag/pol-specific $CD4^+$ T cells producing IFN-γ, TNF-α, and IL2 in SHIV-Ag85B (Exp 1)-inoculated macaques, SHIV-NI-inoculated macaques and control macaques. The cytokine profile in cells was determined by flow cytometry by gating for lymphocytes and $CD4^+$ T cells. PBMCs obtained at 0 weeks and 2 weeks after SHIV89.6P challenge were co-cultured for 6 h with autologous B-LCL cells that had been infected with a recombinant vaccinia virus expressing SIV Gag/pol. **b** Mean percentages of Gag/pol-specific induction of single or multiple cytokines in SHIV-Ag85B (Exp 1)-inoculated macaques, SHIV-NI-inoculated macaques and control macaques after SHIV89.6P challenge. Error bars represent means ± SEM. Statistically significant differences between SHIV-Ag85B (Exp 1) and SHIV-NI were determined by using Student's *t* test.

Supplementary Fig. 8

a

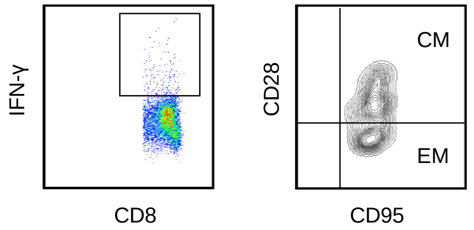

b

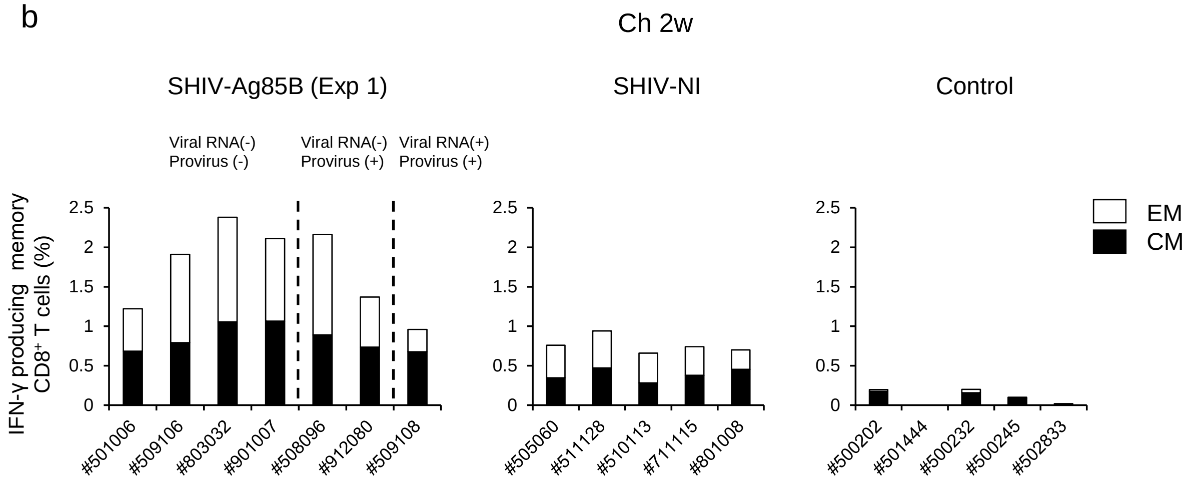

Supplementary Fig. 8 SHIV antigen-specific CD8$^+$ T cell memory subsets. CD8$^+$ T cell memory subsets in SHIV-Ag85B (Exp 1)-inoculated macaques, SHIV-NI-inoculated macaques and control macaques after SHIV89.6P challenge. **a** Gag/pol-specific IFN-γ$^+$ CD8$^+$ T cells are shown in the plot on the left and memory subsets (CD28 and CD95 expression) are shown in the plot on the right. **b** CD8 central memory (CM; CD28$^+$CD95$^+$) and effector memory (EM; CD28$^-$CD95$^+$) responses to Gag/pol as determined by IFN-γ intracellular cytokine analysis. PBMCs obtained at 2 weeks after SHIV89.6P challenge were co-cultured for 6 h with autologous B-LCL cells that had been infected with a recombinant vaccinia virus expressing SIV Gag/pol.

Supplementary Fig. 9

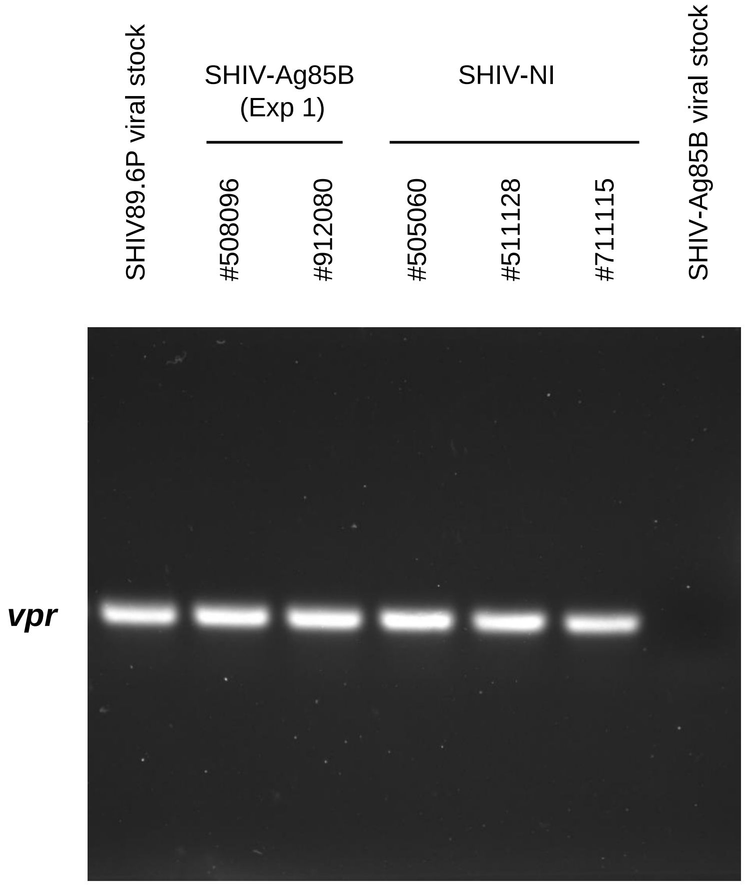

Supplementary Fig. 9 At necropsy, SHIV proviral DNAs in mesenteric lymph nodes of SHIV-Ag85B (Exp 1)-inoculated macaques and SHIV-NI-inoculated macaques were detected by PCR for the SHIV *vpr*-specific region. SHIV89.6P and SHIV-Ag85B cDNAs were used as templates for controls.
