# Supplementary material for: Long-term protective immunity induced by an adjuvant-containing live-attenuated AIDS virus
Source: NPJ Vaccines. 2021 Oct 22;6:124. doi: 10.1038/s41541-021-00386-5 (PMC8536741; doi:10.1038/s41541-021-00386-5)
Supplement: Supplementary file 2 — Reporting Summary [file 41541_2021_386_MOESM2_ESM.pdf]

nature portfolio

Corresponding author(s): Yasuhiro yasutomi

Last updated by author(s): Aug 9, 2021

Reporting Summary

Nature Portfolio wishes to improve the reproducibility of the work that we publish. This form provides structure for consistency and transparency in reporting. For further information on Nature Portfolio policies, see our Editorial Policies and the Editorial Policy Checklist.

Statistics

For all statistical analyses, confirm that the following items are present in the figure legend, table legend, main text, or Methods section.

n/a	Confirmed	
☐	☒	The exact sample size (*n*) for each experimental group/condition, given as a discrete number and unit of measurement
☒	☐	A statement on whether measurements were taken from distinct samples or whether the same sample was measured repeatedly
☐	☒	The statistical test(s) used AND whether they are one- or two-sided<br>*Only common tests should be described solely by name; describe more complex techniques in the Methods section.*
☒	☐	A description of all covariates tested
☐	☒	A description of any assumptions or corrections, such as tests of normality and adjustment for multiple comparisons
☐	☒	A full description of the statistical parameters including central tendency (e.g. means) or other basic estimates (e.g. regression coefficient) AND variation (e.g. standard deviation) or associated estimates of uncertainty (e.g. confidence intervals)
☒	☐	For null hypothesis testing, the test statistic (e.g. *F*, *t*, *r*) with confidence intervals, effect sizes, degrees of freedom and *P* value noted<br>*Give P values as exact values whenever suitable.*
☒	☐	For Bayesian analysis, information on the choice of priors and Markov chain Monte Carlo settings
☒	☐	For hierarchical and complex designs, identification of the appropriate level for tests and full reporting of outcomes
☒	☐	Estimates of effect sizes (e.g. Cohen's *d*, Pearson's *r*), indicating how they were calculated

Our web collection on statistics for biologists contains articles on many of the points above.

Software and code

Policy information about availability of computer code

Data collection	BD FACSDiva version 8.0.1
Data analysis	Flowjo software version 10<br>Graphad Prism software version 7

For manuscripts utilizing custom algorithms or software that are central to the research but not yet described in published literature, software must be made available to editors and reviewers. We strongly encourage code deposition in a community repository (e.g. GitHub). See the Nature Portfolio guidelines for submitting code & software for further information.

Data

Policy information about availability of data

All manuscripts must include a data availability statement. This statement should provide the following information, where applicable:

- Accession codes, unique identifiers, or web links for publicly available datasets
- A description of any restrictions on data availability
- For clinical datasets or third party data, please ensure that the statement adheres to our policy

Raw data on individual macaques shown in the Figures. All data generated in this study are available from the corresponding author upon reasonable request.

Field-specific reporting

Please select the one below that is the best fit for your research. If you are not sure, read the appropriate sections before making your selection.

☒ Life sciences ☐ Behavioural & social sciences ☐ Ecological, evolutionary & environmental sciences

For a reference copy of the document with all sections, see nature.com/documents/nr-reporting-summary-flat.pdf

Life sciences study design

All studies must disclose on these points even when the disclosure is negative.

Sample size	We used 38 adult cynomolgus macaques. Seven cynomolgus macaques were intravenously inoculated with SHIV-Ag85B, five macaques were intravenously inoculated with SHIV-NI and another five macaques were intravenously inoculated with SHIV89.6P (Experiment 1). To evaluate viral replication, a further 7 macaques were inoculated intravenously with SHIV-Ag85B (Experiment 2). To evaluate the protective efficacy of SHIV-Ag85B or SHIV-NI in inoculated macaques, the macaques were challenged intravenously with pathogenic SHIV89.6P at 37 weeks after inoculation. As controls, a further 5 naïve macaques were challenged intravenously with SHIV89.6P. In the adoptive transfer experiment (Experiment 3), 9 naïve macaques were used as SHIV-naïve recipients.
Data exclusions	No data was excluded from any analyis.
Replication	All attempts at replication of virologic and immunologic assays were successful. Multiple time-points were assessed to ensure reproducibility. To study the adjuvant effect of Ag85B in SHIV macaque models, two experiments were performed at different time. At first, Seven cynomolgus macaques were intravenously inoculated with SHIV-Ag85B (first experiment; Exp 1). A further 7 macaques were inoculated intravenously with SHIV-Ag85B (second experiment; Exp 2).
Randomization	Animals were not randomized.
Blinding	The data collection was not blinded. Blinding was not possible as the investigators and the animal handlers were also conducting the all experiments and had to be aware of control and treated groups.

Reporting for specific materials, systems and methods

We require information from authors about some types of materials, experimental systems and methods used in many studies. Here, indicate whether each material, system or method listed is relevant to your study. If you are not sure if a list item applies to your research, read the appropriate section before selecting a response.

Materials & experimental systems

n/a	Involved in the study	
☐	☒	Antibodies
☐	☒	Eukaryotic cell lines
☒	☐	Palaeontology and archaeology
☐	☒	Animals and other organisms
☒	☐	Human research participants
☒	☐	Clinical data
☒	☐	Dual use research of concern

Methods

n/a	Involved in the study	
☒	☐	ChIP-seq
☐	☒	Flow cytometry
☒	☐	MRI-based neuroimaging

Antibodies

Antibodies used	Antibodies used in flow cytometric analysis.<br>CD3-Alexa Fluor 700, clone SP34-2, BD Bioscience, cat 557917, lot 8162839 7146565<br>CD4-PerCP-Cy5.5, clone L200, BD Bioscience, cat 552838, lot 3137892 7038894<br>CD4-APC-H7, clone L200, BD Bioscience, cat 560837, lot 7278806<br>CD8-APC clone DK25, Dako, cat 20038086 20052402<br>CD28-ECD, clone CD28.2, Beckman coulter, cat 6607111, lot 7618049 7618051<br>CD95-FITC, clone DX2, BD Bioscience, cat 556640 lot 6312653<br>IFNγ-PE, clone 4S.BS, BioLegend, cat 502510, lot B136824<br>TNFα-PE-cy7, clone MAB11, BioLegend, cat 502930, lot B247814<br>IL2-PerCP-cy5.5, clone MQ1-17H12, BD Bioscience, cat 560708, lot 3123855
Validation	The specificity and cross-reactivity of the antibodies to non-human primate targets we address in this study have been confirmed by the extensive use and validation of staining patterns in our laboratory with the National Institutes of Health NHP reagent. resource database.

Eukaryotic cell lines

Policy information about cell lines

Cell line source(s)	293T cell, M8166, HSC-F, TZM-bl and CEM×174 cells obtained from NIH AIDS Research and Reference Reagent Program. Cynomolgus macaques PBMCs obtained from Tsukuba primate Research center.
Authentication	None of the cell lines have been authenticated.
Mycoplasma contamination	All cell line tested negative for Mycoplasma contamination.
Commonly misidentified lines (See ICLAC register)	No commonly misidentified cell line were used.

Animals and other organisms

Policy information about studies involving animals; ARRIVE guidelines recommended for reporting animal research

Laboratory animals	We used adult cynomolgus macaques (from Indonesia, Philippines, and Malaysia), all of which were negative for Simian immunodeficiency virus, simian type D retrovirus, simian T-cell lymphotropic virus, simian foamy virus, Epstein-Barr virus, cytomegalovirus, and B virus.
Wild animals	No wild animals were used in this study.
Field-collected samples	No wild field-collected samples were used in this study.
Ethics oversight	These studies were performed in Tsukuba Primate Research Center, National Institutes of Biomedical Innovation, Health and Nutrition (NIBIOHN) after approval by the Committee on the Ethics of Animal Experiments of NIBIOHN in accordance with the guidelines for animal experiments at NIBIOHN.

Note that full information on the approval of the study protocol must also be provided in the manuscript.

Flow Cytometry

Plots

Confirm that:

☒ The axis labels state the marker and fluorochrome used (e.g. CD4-FITC).

☒ The axis scales are clearly visible. Include numbers along axes only for bottom left plot of group (a 'group' is an analysis of identical markers).

☒ All plots are contour plots with outliers or pseudocolor plots.

☒ A numerical value for number of cells or percentage (with statistics) is provided.

Methodology

Sample preparation	Flow Cytometry was performed in PBMCs. PBMCs were isolated from peripheral blood by density gradient centrifugation.
Instrument	BD Bioscience FACSCanto II
Software	BD FACSDiva version 8.0.1, Flowjo software version 10
Cell population abundance	No cell sorts were done.
Gating strategy	The gating strategy for CD4+ and CD8+ T cell counts has been previously published. T cells were gated as Singlets/ Lymphocytes/Viable/CD3+ before CD4+ and CD8+ gating was applied.<br>Okamura, T., et al. Simian immunodeficiency virus SIVmac239 infection and simian human immunodeficiency virus SHIV89.6P infection result in progression to AIDS in cynomolgus macaques of Asian origin. J Gen Virol 97, 3413-3426 (2016).<br><br>The gating strategy for antigen-specific CD4+ and CD8+ T cell responses has been previously published. The cells were gated as Singlets/Lymphocytes/Viable/CD3+ before CD4+ and CD8+ gating was applied. Cytokine positivity was determined by placement of cytokine gates on the control samples and subsequently applying the gates to the Gag/pol-stimulated samples.<br>Yamamoto, T., et al. Virus inhibition activity of effector memory CD8(+) T cells determines simian immunodeficiency virus load in vaccinated monkeys after vaccine breakthrough infection. J Virol 86, 5877-5884 (2012).<br>Yamamoto, T., et al. STING agonists activate latently infected cells and enhance SIV-specific responses ex vivo in naturally SIV controlled cynomolgus macaques. Sci Rep 9, 5917 (2019).

☐ Tick this box to confirm that a figure exemplifying the gating strategy is provided in the Supplementary Information.
